# Fasting glucose, bone area and bone mineral density: a Mendelian randomisation study

**DOI:** 10.1007/s00125-021-05410-w

**Published:** 2021-03-02

**Authors:** Adam Mitchell, Susanna C. Larsson, Tove Fall, Håkan Melhus, Karl Michaëlsson, Liisa Byberg

**Affiliations:** 1 Department of Surgical Sciences, Orthopaedics, Uppsala University, Uppsala, Sweden; 2 Department of Medical Sciences, Molecular Epidemiology, Uppsala University, Uppsala, Sweden; 3 Department of Medical Sciences, Clinical Pharmacogenomics and Osteoporosis, Uppsala University, Uppsala, Sweden

**Keywords:** Bone area, Bone mineral density, Fasting glucose, Mendelian randomisation, Single nucleotide polymorphisms

## Abstract

**Aims/hypothesis:**

Observational studies indicate that type 2 diabetes mellitus and fasting glucose levels are associated with a greater risk for hip fracture, smaller bone area and higher bone mineral density (BMD). However, these findings may be biased by residual confounding and reverse causation. Mendelian randomisation (MR) utilises genetic variants as instruments for exposures in an attempt to address these biases. Thus, we implemented MR to determine whether fasting glucose levels in individuals without diabetes are causally associated with bone area and BMD at the total hip.

**Methods:**

We selected 35 SNPs strongly associated with fasting glucose (*p* < 5 × 10^−8^) in a non-diabetic European-descent population from the Meta-Analyses of Glucose and Insulin-related traits Consortium (MAGIC) (*n* = 133,010). MR was used to assess the associations of genetically predicted fasting glucose concentrations with total hip bone area and BMD in 4966 men and women without diabetes from the Swedish Mammography Cohort, Prospective Investigation of Vasculature in Uppsala Seniors and Uppsala Longitudinal Study of Adult Men.

**Results:**

In a meta-analysis of the three cohorts, a genetically predicted 1 mmol/l increment of fasting glucose was associated with a 2% smaller total hip bone area (−0.67 cm^2^ [95% CI −1.30, −0.03; *p* = 0.039]), yet was also associated, albeit without reaching statistical significance, with a 4% higher total hip BMD (0.040 g/cm^2^ [95% CI −0.00, 0.07; *p* = 0.060]).

**Conclusions/interpretation:**

Fasting glucose may be a causal risk factor for smaller bone area at the hip, yet possibly for greater BMD. Further MR studies with larger sample sizes are required to corroborate these findings.

**Graphical abstract:**

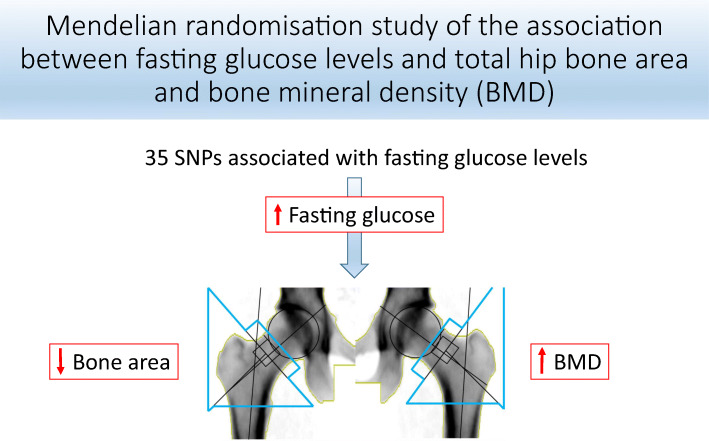

**Supplementary Information:**

The online version contains peer-reviewed but unedited supplementary material available at 10.1007/s00125-021-05410-w.

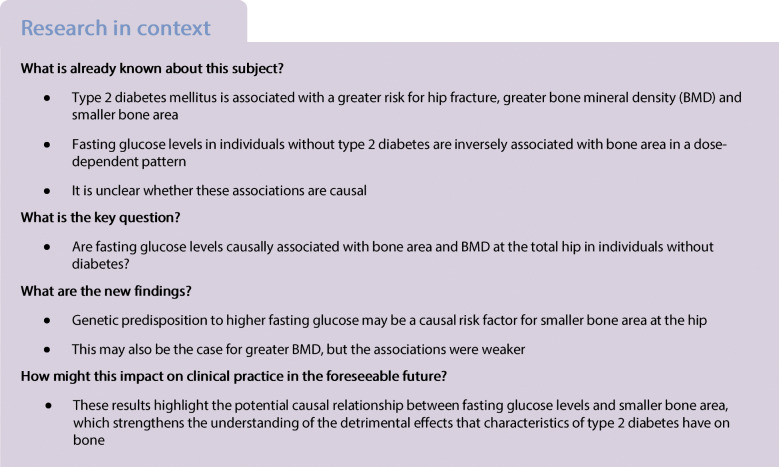



## Introduction

Observational studies have shown type 2 diabetes mellitus to be associated with a greater risk for hip fracture [1, 2], despite also being associated with a greater bone mineral density (BMD) [3]. Type 2 diabetes may therefore have other detrimental effects resulting in a greater fracture risk. Potential mechanisms include weakened bone structure [4], diminished levels of bone turnover with greater bone loss [5], an increased risk of falling [6], or smaller bone area [7]. Bone size is integral for bone strength [8]. Specifically, bone strength is proportional to the fourth power of the radius, such that a doubling in cortex diameter will yield eightfold increments in mechanical resistance to bending and torsional loads [9]. Throughout life, bone expands as a compensation for age-related loss in BMD [10]. We have previously shown, in a cross-sectional study using two Swedish cohorts (Swedish Mammography Cohort - Clinical [SMCC] and Uppsala Longitudinal Study of Adult Men [ULSAM]), that this increase in bone area may be less in individuals with type 2 diabetes [11]. We measured the total hip bone area, but others have also shown smaller bone area at the tibia [12] and radius [7] in those with type 2 diabetes. It is unclear which particular aspects of type 2 diabetes are responsible for the damaging effects on bone, as it is a complex disease characterised by both high blood glucose and complications with insulin action or secretion. In addition to type 2 diabetes, we also showed in our previous study that fasting glucose levels were inversely associated with bone area in a dose-dependent pattern [11].

To determine the causal effects on bone of type 2 diabetes or glucose levels in a non-diabetic population using traditional epidemiological approaches may be difficult due to a number of potential biases, including residual confounding and reverse causation [13]. To conduct a randomised trial where individuals would be randomised to certain levels of fasting glucose for many years to represent type 2 diabetes risk would be unethical and impossible. The Mendelian randomisation (MR) approach uses genetic data as instrumental variables to examine the effects of modifiable risk factors and various disease outcomes, particularly in observational data settings when confounding is a major concern [14]. Moreover, because allele assignment at meiosis is random and precedes the onset of disease, the risk of confounding is limited and there is no concern for the possibility of reverse causation [15]. Previous MR studies indicate that genetically determined fasting glucose levels [16] and type 2 diabetes risk [16, 17] increases BMD at the femoral neck, but no association was seen with risk of any fracture among adults >18 years [17].

No MR study to date has addressed the effect of fasting glucose on bone area, one potential mechanism underlying the association between type 2 diabetes and hip fracture. Several studies indicate that bone area at the hip is linked with the risk of hip fracture [18, 19], and it is therefore of clinical importance to investigate whether the observational association between higher fasting glucose concentrations and a smaller bone area at the hip—previously presented by our research group [11]—is causal. As the genetic instruments available for glucose concentrations were established among individuals without diabetes [20], and the genetically increased glucose concentrations will influence the outcome independent of type 2 diabetes status [21], we implemented an MR approach to determine whether fasting glucose levels are causally associated with total hip bone area, and to verify the association with total hip BMD, in individuals without diabetes in three Swedish cohorts. The observational associations were based on two of these three cohorts [11].

## Methods

### Study samples

All study populations were restricted to individuals without diabetes in the three Swedish cohorts detailed below. We defined diabetes according to the ADA and WHO criteria: fasting plasma glucose concentrations ≥7.0 mmol/l and/or self-reported diabetes with or without treatment with oral hypoglycaemic agents or insulin.

### SMCC

The Swedish Mammography Cohort (SMC) was established during 1987–1990. Between November 2003 and October 2009, a randomly selected sub-cohort (SMCC) of 5022 women living in the city of Uppsala, Sweden, underwent dual energy x-ray absorptiometry (DXA) measurements, provided morning fasting blood samples, had height and weight measurements taken, and completed a medical and lifestyle questionnaire [22]. Of these, 3945 women had complete information on fasting glucose, diabetes status, genetic data and DXA measurements and were thus included in our analyses. Genotyping in the SMCC was performed using the Illumina GSAMD-24v1-0_20011747_A1 BeadChip, USA and SNPs were imputed up to Haplotype Reference Consortium (HRC) v1.1 and 1000 Genomes project phase 3. The results were then analysed using the software GenomeStudio 2.0.3 from Illumina, USA. The sample success rate was ≥98%. The SMCC is managed by the Swedish Infrastructure for Medical Population-based Life-course and Environmental Research (www.simpler4health.se).

### Prospective investigation of the vasculature of Uppsala seniors

Between 2001 and 2004, all 70-year-old residents of Uppsala, Sweden, were invited to participate in a health survey and clinical assessment [23]. Of 2025 invited, 1016 (50.2%) participated in the baseline assessment. From these, 691 participants had complete information on fasting blood glucose (converted to plasma concentrations) [24], diabetes status, genetic data and DXA measurements and were included in our analyses. Genotyping in the Prospective Investigation of the Vasculature of Uppsala Seniors (PIVUS) was performed using Illumina OmniExpress+Metabochip, USA, quality controlled and imputed up to the HRC panel using the software IMPUTE (https://mathgen.stats.ox.ac.uk/impute/impute_v1_html). The sample success rate was 98.8% and the reproducibility 100% according to duplicate analysis of 2.4% of the genotypes.

### ULSAM

In 1970, all men born between 1920 and 1924, living in the county of Uppsala, Sweden, were invited to a take part in a health survey [25]. The men who participated were regularly re-examined, and the current analyses were based on the fifth examination cycle in 2003–2005, when 952 men were invited for examination and 526 of them were examined (mean age 82 years). Of these men, 360 had complete information on fasting glucose, diabetes status, genetic data and DXA measurements and were thus included in our analyses. Genotyping was performed using Illumina Omni2.5+Metabochip and GenomeStudio 2010.3, USA and imputed up to the HRC panel using the software IMPUTE. The sample success rate was ≥99%, minor allele frequency (MAF <5%) or ≥95% (MAF ≥5%).

This study complies with the Declaration of Helsinki. The ethics committee of Uppsala University approved the studies (ethical approval numbers 2010/0148-32 [Stockholm] and 2019-02125 [Uppsala]). All participants provided their informed consent.

### Bone area and BMD

**Fig. 1:**
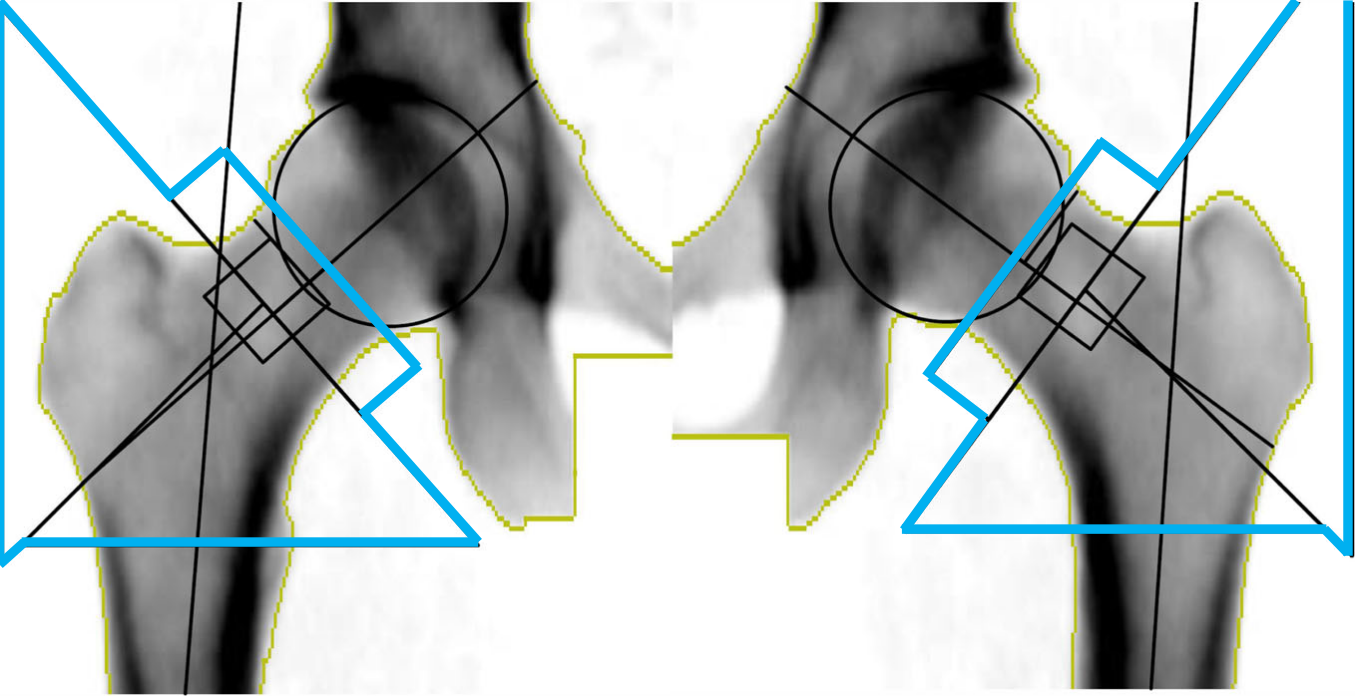
DXA image of dual femur and the total hip in an adult male. The total area within the blue lines defines the total hip area ROI. This is the standard output from the Lunar Prodigy DXA scanner [11]

Bone area (cm^2^) and BMD (g/cm^2^) of the total hip and femoral shaft were measured by DXA (DPX Prodigy, Lunar, Madison, WI, USA). All measurements in all three cohorts were performed on the same DXA machine by the same experienced and accredited DXA x-ray nurse. The hip was set in a standard position by a fixed position of the knee, ankle and foot, to ensure that area did not vary due to rotational differences, and each scan was checked before it was accepted. The total hip area region of interest (ROI) was defined as the total area within the blue lines, corresponding to the femoral neck, Ward’s area, trochanter, and femoral shaft ROIs (Fig. 1) [11]. The ROI was adjusted to the same location for each participant if needed (< 0.5% of scans). The precision error from the DXA was <1% for BMD and bone area at the total hip. To quantify differences in bone area and BMD, we calculated a percentage difference by dividing the β estimate generated from the meta-analysed inverse-variance weighted (IVW) regression models by the mean value of either bone area or BMD multiplied by 100.

### SNP selection

As instrumental variables, we selected the 36 SNPs associated with fasting glucose concentrations at a genome-wide significance threshold (*p* < 5× 10^−8^) in a population without diabetes of European descent (*n* = 133,010) from the Meta-Analyses of Glucose and Insulin-related traits Consortium (MAGIC) [20]. One SNP (rs10747083) was associated with fasting glucose in the opposite direction compared with that in MAGIC in all three included Swedish cohorts and was excluded from subsequent analysis as it was not deemed a robust instrument, leaving 35 SNPs as instrumental variables in the present analyses. All 35 SNPs were available in the three cohorts and were independent (linkage disequilibrium R^2^ < 0.01 in European population).

### MR analysis

**Fig. 2:**
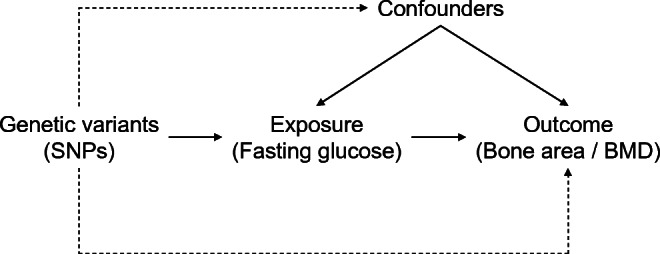
The instrumental variables assumptions for MR. The three assumptions are: (1) the genetic variants are robustly associated with the exposure; (2) they are not associated with confounders of the exposure–outcome relationship; and (3) they have no association with the outcome except through their association with the exposure. The dashed lines represent pathways that violate the assumptions

The MR approach was used to obtain quantitative estimates of the causal effects of fasting glucose on total hip bone area and BMD, based on the assumptions that the genetic variants used as instrumental variables: (1) are associated with the exposure (fasting glucose); (2) are not associated with any confounders of the exposure–outcome association; and (3) are associated with bone area and BMD through the exposure only and not through any alternative causal pathway ensuring a lack of pleiotropy (Fig. 2).

Linear regression models, adjusted for age and genetic principal components (SMCC *n* = 10, PIVUS *n* = 2, ULSAM *n* = 4), were applied to estimate the association between each SNP and bone area and BMD at the total hip. In the primary analysis, the SNP–glucose and SNP–bone outcome β coefficients were used to compute estimates of the associations of fasting glucose with the bone outcomes using the IVW method [26], first using fixed effects and then with random effects [27]. The MR estimates (β coefficients and standard errors) for the associations between genetically predicted fasting glucose and the outcomes computed from each of the three cohorts were then combined in a meta-analysis using the metan package for Stata (https://raw.github.com/remlapmot/mrrobust/master/).

To explore the robustness of the MR results we conducted analysis using the weighted median, which can provide a consistent estimate of the causal effect even when up to 50% of the genetic variants are invalid instruments [28]. We applied MR-Egger regression [29] methods using the mrrobust package [30] to identify and control for bias due to directional pleiotropy. Pleiotropy was evaluated based on the intercept obtained from the MR-Egger analysis [31]. To identify any potential outliers and examine the extent of horizontal pleiotropy, we applied the MR-Pleiotropy RESidual Sum and Outlier (PRESSO) method [32] using the MR-PRESSO package in R (https://github.com/rondolab/MR-PRESSO).

In sensitivity analyses, we used multivariable MR analysis to adjust for genetically predicted height [33] and BMI because of the known effects of height and BMI [34] on bone size and diabetes risk, and also removed SNP (rs7651090, for human gene *IGF2BP2*) due to the known effects of IGF binding proteins on bone health. We then performed the above main analyses also including those with type 2 diabetes in our cohorts (total *n* = 4234 in SMCC, 783 in PIVUS, 443 in ULSAM) and using sex-specific β estimates for the associations of the SNPs with fasting glucose (accessed, 20 August 2020 from https://www.magicinvestigators.org/downloads/) and total hip bone area and BMD in our cohorts. A weighted genetic risk score (wGRS) was generated using the 35 SNPs and the β estimates from the MAGIC consortium genome-wide association study data, and we conducted a one-sample MR using the wGRS as the instrumental variable to estimate its association with bone area and BMD using the Wald ratio method (95% CI calculated using the delta method) and additionally with further adjustment for BMI and height. Statistical analyses were performed in Stata MP 15 (StataCorp, College Station, TX, USA) and R, partly using resources provided by SNIC-SENS (a SNIC project with the purpose of providing secure handling of sensitive data [such as human genomic data] to the research community) through the Uppsala Multidisciplinary Center for Advanced Computational Science (UPPMAX). A *p* value of <0.05 was considered statistically significant.

## Results

The characteristics of participants in each of the three Swedish cohorts are presented in Table 1. The mean weight, height, bone area and BMD were higher in both PIVUS and ULSAM compared with SMCC, as men were present in these cohorts, whereas SMCC comprised only women. Fasting glucose levels were also higher in PIVUS and ULSAM which may be explained by the older age in these cohorts compared with SMCC.

**Table 1:** Characteristics of the study population in the three cohorts by sex

Characteristic	SMCC (*n* = 3945)	PIVUS		ULSAM (*n* = 360)
		Women (*n* = 359)	Men (*n* = 332)	
Age (years)	67.18 ± 6.55	70.24 ± 0.28	70.10 ± 0.13	81.69 ± 0.98
BMI (kg/m^2^)	25.75 ± 4.20	26.67 ± 4.40	26.90 ± 3.60	25.71 ± 3.25
Weight (kg)	68.87 ± 11.73	69.36 ± 12.47	82.40 ± 12.21	76.75 ± 10.59
Height (cm)	163.54 ± 6.05	161.63 ± 5.54	175.64 ± 6.35	172.71 ± 5.78
Total hip bone area (cm^2^)	32.50 ± 2.17	32.91 ± 2.10	38.75 ± 2.35	39.16 ± 2.70
Total hip BMD (g/cm^2^)	0.91 ± 0.13	0.87 ± 0.13	1.02 ± 0.15	0.96 ± 0.16
Fasting glucose (mmol/l)	5.15 ± 0.53	5.50 ± 0.55	5.60 ± 0.60	5.53 ± 0.58

Data are presented as mean ± SD

In PIVUS, fasting glucose samples were converted from whole blood to plasma concentrations

**Fig. 3:**
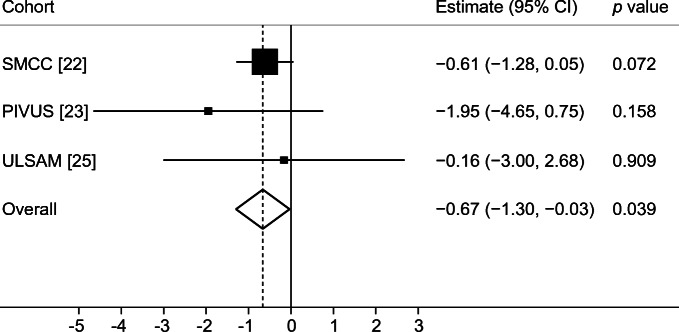
Meta-analysis of glucose variants to bone area in SMCC, PIVUS and ULSAM

**Fig. 4:**
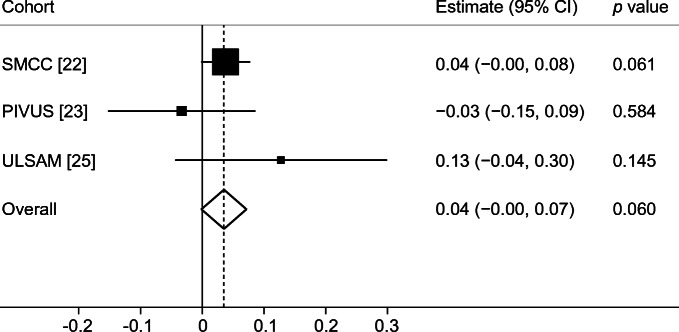
Meta-analysis of glucose variants to BMD in SMCC, PIVUS and ULSAM

**Fig. 5:**
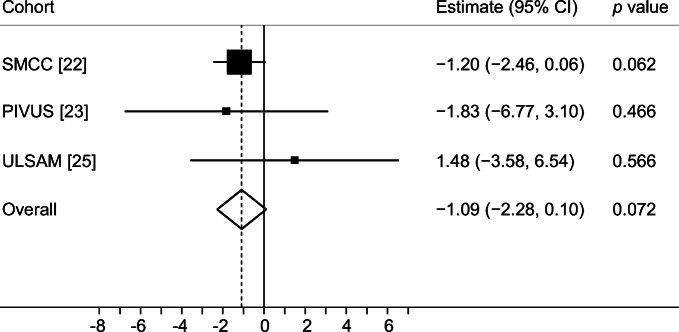
Meta-analysis of glucose variants to bone area in SMCC, PIVUS and ULSAM from MR-Egger test

**Fig. 6:**
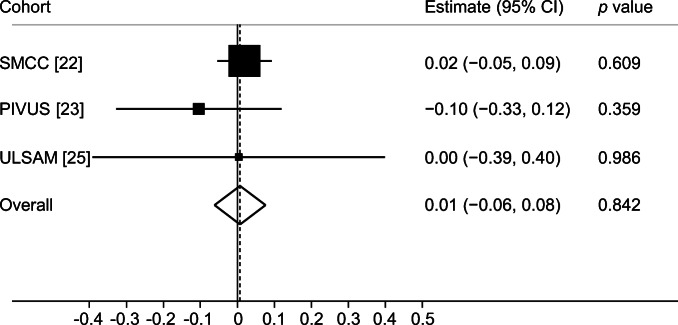
Meta-analysis of glucose variants to BMD in SMCC, PIVUS and ULSAM from MR-Egger test

In the SMCC, PIVUS and ULSAM cohorts, the 35 SNPs used in our analyses together explained 4% (adjusted r^2^ = 0.045), 4% (adjusted r^2^ = 0.035) and 12% (adjusted r^2^ = 0.119), respectively, of the variance in fasting glucose concentrations in participants without diabetes. The association of each SNP with bone area and BMD in each cohort, along with the β estimates for the associations of the SNPs with fasting glucose concentrations, can be found in electronic supplementary material (ESM) Figs 1–6 and ESM Tables 1–3. There was low sample overlap (0.79%) with PIVUS and ULSAM. SMCC, the largest cohort, is not included in MAGIC.

In conventional MR analysis, a 1 mmol/l higher genetically predicted fasting glucose concentration was associated with 2% smaller total hip bone area (−0.67 cm^2^ [95% CI −1.30, −0.03; *p* = 0.039]) based on meta-analysis of estimates from the three cohorts using the fixed effects IVW method (Fig. 3). In contrast to bone area, a 1 mmol/l higher genetically predicted fasting glucose concentration was associated, albeit without reaching statistical significance, with 4% higher total hip BMD (0.040 g/cm^2^ [95% CI −0.00, 0.07; *p* = 0.060]) in meta-analysis of estimates from the three cohorts (Fig. 4), although the precision in the estimate was low. Results were consistent in sensitivity analyses using the random effects IVW and weighted median methods (ESM Figs 7, 8).

We tested for pleiotropy using MR-Egger and MR-PRESSO. The MR-Egger analysis did not provide evidence of pleiotropy in the analysis of bone area or BMD (Table 2) and the causal estimate was in the same direction as the primary analysis (Figs 5, 6). The MR-PRESSO method did not detect any outliers, but the global test *p* value was 0.0129 in the BMD analysis based on ULSAM data indicating there may be some horizontal pleiotropy in that specific analysis. We found no evidence of heterogeneity in the primary analysis using the IVW method (ESM Table 4). Additional sensitivity analysis adjusting for the genetic effect of height and removing SNP rs7651090 did not affect the results essentially (ESM Figs 9–12) and neither did BMI (ESM Figs 13, 14). Including also those with type 2 diabetes in our cohorts did not change the interpretation of our results (ESM Figs 15, 16). Performing the analysis using sex-specific β estimates gave similar results to the main analysis (ESM Figs 17, 18). In the one-sample MR analysis using the wGRS, estimates were once again in the same direction as the primary analysis (ESM Figs 19, 20) even with further adjustment for height and BMI (ESM Figs 21, 22). The resulting F-statistics were 196, 40 and 50 in SMCC, PIVUS and ULSAM, respectively (ESM Table 5).

**Table 2:** MR-Egger tests for the presence of pleiotropy affecting the assessment of the effects of fasting glucose on the outcomes bone area and BMD of the total hip

Outcome	Cohort	β	SE	*p* value
Total hip bone area	SMCC	0.197227	0.179006	0.271
Total hip bone area	PIVUS	−0.003845	0.0691082	0.956
Total hip bone area	ULSAM	−0.055295	0.0718004	0.441
Total hip BMD	SMCC	0.0006225	0.0010413	0.550
Total hip BMD	PIVUS	0.0023567	0.0031256	0.451
Total hip BMD	ULSAM	0.0041666	0.0056075	0.457

## Discussion

To the best of our knowledge, this is the first MR study using multiple genetic variants for fasting glucose and analysing their effect on bone area and BMD. Among men and women without diabetes in three Swedish cohorts, we found evidence that genetically increased fasting glucose levels were associated with smaller total hip bone area. The previously observed association with a greater BMD [16] was also observed in our study. We observed no evidence for directional pleiotropy. These results are partly based on the same population from SMCC and ULSAM where we previously observed that type 2 diabetes and higher fasting glucose levels were associated with smaller bone area and greater BMD at the total hip [11].

Major strengths of the MR approach are that reverse causation bias is avoided because genetic variants are fixed at conception, and confounding is reduced by the use of genetic variants as proxies for the exposure representing the lifelong exposure of fasting glucose concentrations [35]. Our study population consisted of both men and women, and all DXA measurements in all three cohorts were taken by the same x-ray nurse using the same DXA scanner, thereby increasing the reliability and reducing variance in our outcome measure. Magnification error from DXA scans can have direct effects on estimated bone area [36]. The Lunar Prodigy DXA scanner used in this study reduces the substantial magnification error by using a narrow fan-beam along the axis of measurement compared with other scanners which use a wide-angle fan-beam [36, 37]. This reduces the impact of an individual’s body weight which may otherwise affect the bone area measurement. The narrow fan-beam, high resolution, automatic location of the bone, and centring of the scan around the bone providing precise automatic edge detection combine to give an improved measurement that does not require any scout scans and is less dependent on the exact positioning of the femur in the beam [36, 37]. In all cohorts, we were able to clinically categorise individuals as having diabetes based on fasting glucose samples in combination with self-reported diagnosis and medication use, so that they could be excluded from the main analysis. The 35 genetic variants increase the fasting glucose levels, explaining 4–12% of the variance in our studies, and will therefore influence the outcome even if the type 2 diabetes status remains fixed for all participants in the analysis [21]. For a genetic instrument to be valid for a dichotomous exposure, such as type 2 diabetes, there needs to be a strict stepwise threshold at the cut-off point, and the interpretation of such an exposure is less straightforward than that of a continuous exposure [21]. Furthermore, the genetic instruments for type 2 diabetes may be influenced by treatment, and genetic instruments for insulin explain a very low proportion of the variance in insulin concentrations [20]. Although heritability for type 2 diabetes is high, using genetic variants for fasting glucose concentrations as an instrument may therefore provide more specific insight into the mechanisms of glucose on bone area, also when explored in a population without diabetes [21]. Pleiotropic effects of the type 2 diabetes instrument is a concern since it includes obesity-related SNPs, which increase the risk of diabetes through increasing BMI. For fracture-related outcomes, this could pose an additional problem by effects acting in opposing directions, as suggested by prior results showing type 2 diabetes to be associated with higher fracture risk and a high body weight with a lower fracture risk. Nevertheless, conducting the analysis among all participants showed results of the same direction and magnitude as the main analysis, indicating no large influence of potential collider bias after restriction to those with type 2 diabetes. The main limitation of our study is the limited study size, since MR analyses generally require large numbers of individuals. However, we were able to observe statistically significant results for the association between fasting glucose and bone area, and results were consistent using both two-sample MR and one-sample MR designs. We hope future studies and consortia will explore and present results also for bone area. A previous two-sample MR study [16], using summary level data of BMD-associated genetic variants from European and East Asian ancestry [38] among up to 83,894 individuals, reported that genetically increased fasting glucose increased femoral neck BMD and, albeit without reaching statistical significance, lumbar spine BMD. The authors suggested that diabetes may have different effects on cortical and trabecular bone. The total hip, used in our study, consisted of both cortical and trabecular bone and we found similar effects on BMD in that area, although lacking statistical power due to a small sample size. Another MR study found no association of genetically increased fasting glucose and type 2 diabetes on the risk of any fracture among adults >18 years [17]. Fragility fractures such as hip fractures occurring among older individuals may have a different aetiology than other types of fractures occurring among younger individuals, and to separate by fracture type may be crucial. Unfortunately, valid hip fracture information is, to date, lacking from large consortia. Inferring causality from MR analyses relies on several assumptions, one being the assumption that the genetic variants used as instruments are strongly associated with the risk factor. In a two-sample MR setting we used several genetic variants strongly associated with fasting glucose (*p* < 5 × 10^−8^) in a previous meta-analysis of genome-wide association studies of non-diabetic individuals [20], which would ensure that any bias from weak instruments is towards the null [39] and therefore increase the statistical power [26]. The instrument explained 4–12% of the variation in fasting glucose concentrations in our populations, which is high compared with many traits. Further, results were in the same direction as in our main analysis when we applied the weighted median method, where the estimate remains consistent when up to 50% of the SNPs are invalid instruments [27], suggesting robust estimates. The overlap between our outcome cohorts and the MAGIC consortium data was very low and the F-statistics for our individual cohorts were high when assessing the association between the wGRS and fasting glucose levels, indicating that any bias would be towards the null.

MR analyses further rely on the assumption of absence of pleiotropy, which can occur when a genetic instrument (SNP) affects multiple phenotypes [40]. Dependent on the type of pleiotropy present, it can lead to biased estimates. Horizontal pleiotropy occurs when a genetic variant affects more than one phenotype on separate pathways [41], whereas vertical pleiotropy, also known as mediated pleiotropy, occurs when a genetic variant affects other phenotypes downstream from the exposure, on the causal pathway to the outcome [42]. In our case, type 2 diabetes might be one such mediating phenotype, being downstream of fasting glucose and also associated with bone phenotypes. Horizontal pleiotropy can lead to bias in an MR study [43] whereas vertical pleiotropy is of less concern. We cannot entirely exclude the possibility that the SNPs used as instruments in the present study may affect bone area and BMD through mechanisms other than their effects on fasting glucose. However, we did not find any evidence of horizontal or vertical pleiotropy using the MR-Egger approach. Our results also remained consistent when we adjusted for the genetic effects of height [33] and BMI [34] in a sensitivity analysis. Height has been shown to directly affect both bone size and the risk of diabetes [44].

We did not find any evidence of SNPs being outliers or effect change caused by any SNP outliers, evaluated using MR-PRESSO [32], although there may have been some pleiotropy in the ULSAM BMD analysis. Due to the known effects of IGF binding proteins on bone health [45], we removed SNP (rs7651090, for human gene *IGF2BP2*) in an additional sensitivity analysis but this did not affect our estimates. Another potential source of bias in MR analyses is population stratification and population heterogeneity [46], but this was reduced in our study because our genetic instruments and outcomes came from European populations and all our outcome populations were based in Sweden. We also adjusted for genetic principal components. However, this may limit the generalisability of our results to other ethnicities.

Bone size increases with advancing age via periosteal apposition to compensate for the losses in BMD in order to preserve bone strength [47]. The bending strength of bone and resistance to fracture is strongly related to bone diameter and small differences in area will result in large differences in strength [48]. It has been reported that increases in bone cortex diameter increases resistance to bending, torsional and compressive loads [49]. This suggests that the smaller bone area, seen with higher glucose concentrations, will yield a comparably lower bending resistance and strength, resulting in a bone that is more susceptible to fracture. The risk of incident hip fracture in older white women from the USA has been shown to be higher with lower composite indices of femoral neck strength [8].

The relationship between bone and energy metabolism is complex and may be bidirectional. A previous study found that each one SD increase in genetically estimated heel BMD (equivalent to 0.14 g/cm^2^) was associated with an 8% higher risk of type 2 diabetes, assessed using an MR approach [50]. Consequently, it could be of interest to conduct a bidirectional MR study, although a major limitation is the low number of genetic variants reported for bone area. A study with large power could assess this direction of association.

By combining data from three separate cohorts in a two-sample MR design, our study provided evidence that fasting glucose may be a causal risk factor for smaller bone area at the hip. We found suggestive evidence that fasting glucose may also lead to increased BMD. If other larger cohorts or consortia with DXA measurements of the hip, such as the UK Biobank, presented their bone area measurements, future MR studies could utilise the larger sample sizes required to corroborate these findings.

## Supplementary Information

ESM (PDF 3709 kb)

## Data Availability

The data codes used to generate the results in this study are available from the corresponding author upon reasonable request. Data are not freely available, but the cohorts can be contacted to request access. SMCC: https://www.simpler4health.se/; PIVUS: https://www.medsci.uu.se/pivus; ULSAM: https://www.pubcare.uu.se/ulsam/

## Abbreviations

BMD: Bone mineral density
DXA: Dual energy x-ray absorptiometry
HRC: Haplotype Reference Consortium
IVW: Inverse-variance weighted
MAF: Minor allele frequency
MAGIC: Meta-Analyses of Glucose and Insulin-related traits Consortium
MR: Mendelian randomisation
PIVUS: Prospective Investigation of the Vasculature of Uppsala Seniors
ROI: Region of interest
SMC: Swedish Mammography Cohort
SMCC: Swedish Mammography Cohort - Clinical
ULSAM: Uppsala Longitudinal Study of Adult Men
wGRS: Weighted genetic risk score
