# Supplementary material for: Fasting glucose, bone area and bone mineral density: a Mendelian randomisation study
Source: Diabetologia. 2021 Mar 2;64(6):1348–57. doi: 10.1007/s00125-021-05410-w (PMC8099809; doi:10.1007/s00125-021-05410-w)
Supplement: Supplementary file 1 — (PDF 3709 kb) [file 125_2021_5410_MOESM1_ESM.pdf]

ESM Table 1 Glucose SNP associations from MAGIC and SMCC

trait	hg19_coordinate	snp	Gene	effall	otherall	betatrait	setrait	estimate	stderr	P
glucose	09:22134094	rs10811661	*CDKN2B*	T	C	0.024	0.003	-0.0008	0.0156	0.9589
glucose	11:92708710	rs10830963	*MTNR1B*	G	C	0.078	0.002	0.0720	0.0131	0.0000
glucose	10:113042093	rs10885122	*ADRA2A*	G	T	0.027	0.003	0.0027	0.0176	0.8788
glucose	15:62433962	rs11071657	*VPS13C/C2CD4A/B*	A	G	0.010	0.002	0.0186	0.0120	0.1218
glucose	08:118185733	rs11558471	*SLC30A8*	A	G	0.029	0.002	0.0489	0.0126	0.0001
glucose	11:72432985	rs11603334	*ARAP1*	G	A	0.019	0.003	0.0437	0.0151	0.0038
glucose	11:45873091	rs11605924	*CRY2*	A	C	0.020	0.002	0.0199	0.0117	0.0899
glucose	13:28487599	rs11619319	*PDX1*	G	A	0.019	0.002	0.0476	0.0144	0.0010
glucose	03:123065778	rs11708067	*ADCY5*	A	G	0.023	0.003	0.0108	0.0141	0.4450
glucose	03:49455330	rs11715915	*AMT*	C	T	0.012	0.002	0.0008	0.0124	0.9459
glucose	03:170717521	rs11920090	*SLC2A2*	T	A	0.026	0.003	0.0316	0.0178	0.0765
glucose	09:111680359	rs16913693	*IKBKAP*	T	G	0.043	0.007	0.0914	0.0294	0.0019
glucose	11:61571478	rs174550	*FADS1*	T	C	0.019	0.002	0.0143	0.0122	0.2412
glucose	06:7213200	rs17762454	*RREB1*	T	C	0.014	0.002	0.0087	0.0139	0.5295
glucose	07:15064309	rs2191349	*DGKB/TMEM195*	T	G	0.029	0.002	0.0385	0.0118	0.0011
glucose		rs2302593	*GIPR*	C	G	0.014	0.002	0.0295	0.0118	0.0124
glucose	12:56865338	rs2657879	*GLS2*	G	A	0.016	0.003	-0.0142	0.0160	0.3762
glucose	01:214159256	rs340874	*PROX1*	C	T	0.013	0.002	0.0445	0.0118	0.0002
glucose	14:100839261	rs3783347	*WARS*	G	T	0.017	0.003	0.0062	0.0138	0.6546
glucose	09:139256766	rs3829109	*LOC728489*	G	A	0.017	0.003	0.0154	0.0128	0.2260
glucose	10:114756041	rs4506565	*TCF7L2*	T	A	0.021	0.002	0.0285	0.0131	0.0301
glucose	07:44235668	rs4607517	*GCK*	A	G	0.057	0.003	0.0674	0.0170	0.0001
glucose	05:95539448	rs4869272	*PCSK1*	T	C	0.018	0.002	0.0038	0.0126	0.7652
glucose	02:169763148	rs560887	*G6PC2*	C	T	0.071	0.002	0.0872	0.0128	0.0000
glucose	13:33554302	rs576674	*KL*	G	A	0.017	0.003	-0.0056	0.0167	0.7393

glucose	20:39743905	rs6072275	*TOP1*	A	G	0.016	0.003	0.0111	0.0151	0.4648
glucose	20:22557099	rs6113722	*FOXA2*	G	A	0.035	0.005	0.0588	0.0328	0.0726
glucose	07:50791579	rs6943153	*GRB10*	T	C	0.015	0.002	0.0249	0.0128	0.0526
glucose	03:185513392	rs7651090	*IGF2BP2*	G	A	0.013	0.002	0.0168	0.0131	0.2010
glucose	05:76425867	rs7708285	*ZBED3*	G	A	0.015	0.003	0.0304	0.0136	0.0255
glucose	02:27741237	rs780094	*GCKR*	C	T	0.027	0.002	0.0278	0.0124	0.0253
glucose	09:4292152	rs7867224	*GLIS3*	A	G	0.013	0.002	0.0081	0.0117	0.4912
glucose	11:47336320	rs7944584	*MADD*	A	T	0.023	0.002	0.0405	0.0133	0.0024
glucose	06:20686996	rs9368222	*CDKAL1*	A	C	0.014	0.002	0.0208	0.0135	0.1240
glucose	08:9177732	rs983309	*PPP1R3B*	T	G	0.026	0.003	0.0578	0.0185	0.0018

ESM Table 2 Glucose SNP associations from MAGIC and PIVUS

trait	hg19_coordinate	snp	Gene	effall	otherall	betatrait	setrait	estimate	stderr	p
glucose	09:22134094	rs10811661	*CDKN2B*	T	C	0.024	0.003	0.0093	0.0322	0.7726
glucose	11:92708710	rs10830963	*MTNR1B*	G	C	0.078	0.002	0.0597	0.0277	0.0312
glucose	10:113042093	rs10885122	*ADRA2A*	G	T	0.027	0.003	0.0314	0.0374	0.4014
glucose	15:62433962	rs11071657	*VPS13C/C2CD4A/B*	A	G	0.010	0.002	-0.0062	0.0249	0.8051
glucose	08:118185733	rs11558471	*SLC30A8*	A	G	0.029	0.002	0.0141	0.0259	0.5852
glucose	11:72432985	rs11603334	*ARAP1*	G	A	0.019	0.003	0.0345	0.0332	0.2993
glucose	11:45873091	rs11605924	*CRY2*	A	C	0.020	0.002	0.0207	0.0249	0.4052
glucose	13:28487599	rs11619319	*PDX1*	G	A	0.019	0.002	0.0417	0.0295	0.1580
glucose	03:123065778	rs11708067	*ADCY5*	A	G	0.023	0.003	0.0691	0.0304	0.0234
glucose	03:49455330	rs11715915	*AMT*	C	T	0.012	0.002	0.0359	0.0260	0.1672
glucose	03:170717521	rs11920090	*SLC2A2*	T	A	0.026	0.003	0.0281	0.0375	0.4540
glucose	09:111680359	rs16913693	*IKBKAP*	T	G	0.043	0.007	0.0245	0.0599	0.6831
glucose	11:61571478	rs174550	*FADS1*	T	C	0.019	0.002	0.0181	0.0249	0.4668
glucose	06:7213200	rs17762454	*RREB1*	T	C	0.014	0.002	-0.0089	0.0293	0.7608
glucose	07:15064309	rs2191349	*DGKB/TMEM195*	T	G	0.029	0.002	0.0123	0.0248	0.6182
glucose		rs2302593	*GIPR*	C	G	0.014	0.002	0.0401	0.0244	0.1011
glucose	12:56865338	rs2657879	*GLS2*	G	A	0.016	0.003	0.0217	0.0342	0.5257
glucose	01:214159256	rs340874	*PROX1*	C	T	0.013	0.002	0.0353	0.0245	0.1493
glucose	14:100839261	rs3783347	*WARS*	G	T	0.017	0.003	0.0293	0.0279	0.2949
glucose	09:139256766	rs3829109	*LOC728489*	G	A	0.017	0.003	0.0558	0.0259	0.0313
glucose	10:114756041	rs4506565	*TCF7L2*	T	A	0.021	0.002	0.0331	0.0292	0.2576
glucose	07:44235668	rs4607517	*GCK*	A	G	0.057	0.003	0.1076	0.0380	0.0048
glucose	05:95539448	rs4869272	*PCSK1*	T	C	0.018	0.002	0.0178	0.0265	0.5023
glucose	02:169763148	rs560887	*G6PC2*	C	T	0.071	0.002	0.0828	0.0275	0.0027
glucose	13:33554302	rs576674	*KL*	G	A	0.017	0.003	0.0361	0.0332	0.2765

glucose	20:39743905	rs6072275	*TOP1*	A	G	0.016	0.003	-0.0035	0.0326	0.9133
glucose	20:22557099	rs6113722	*FOXA2*	G	A	0.035	0.005	-0.0840	0.0637	0.1881
glucose	07:50791579	rs6943153	*GRB10*	T	C	0.015	0.002	0.0118	0.0254	0.6432
glucose	03:185513392	rs7651090	*IGF2BP2*	G	A	0.013	0.002	0.0053	0.0275	0.8460
glucose	05:76425867	rs7708285	*ZBED3*	G	A	0.015	0.003	-0.0216	0.0270	0.4242
glucose	02:27741237	rs780094	*GCKR*	C	T	0.027	0.002	0.0074	0.0266	0.7820
glucose	09:4292152	rs7867224	*GLIS3*	A	G	0.013	0.002	0.0156	0.0248	0.5315
glucose	11:47336320	rs7944584	*MADD*	A	T	0.023	0.002	-0.0022	0.0286	0.9378
glucose	06:20686996	rs9368222	*CDKAL1*	A	C	0.014	0.002	0.0294	0.0286	0.3042
glucose	08:9177732	rs983309	*PPP1R3B*	T	G	0.026	0.003	0.1178	0.0408	0.0040

ESM Table 3 Glucose SNP associations from MAGIC and ULSAM

trait	hg19_coordinate	snp	Gene	effall	otherall	betatrait	setrait	estimate	stderr	p
glucose	09:22134094	rs10811661	*CDKN2B*	T	C	0.024	0.003	0.0425	0.0524	0.4179
glucose	11:92708710	rs10830963	*MTNR1B*	G	C	0.078	0.002	0.0973	0.0476	0.0417
glucose	10:113042093	rs10885122	*ADRA2A*	G	T	0.027	0.003	0.1119	0.0632	0.0773
glucose	15:62433962	rs11071657	*VPS13C/C2CD4A/B*	A	G	0.010	0.002	0.0843	0.0407	0.0391
glucose	08:118185733	rs11558471	*SLC30A8*	A	G	0.029	0.002	0.1209	0.0446	0.0069
glucose	11:72432985	rs11603334	*ARAP1*	G	A	0.019	0.003	0.1159	0.0523	0.0274
glucose	11:45873091	rs11605924	*CRY2*	A	C	0.020	0.002	0.0636	0.0401	0.1139
glucose	13:28487599	rs11619319	*PDX1*	G	A	0.019	0.002	-0.0744	0.0491	0.1303
glucose	03:123065778	rs11708067	*ADCY5*	A	G	0.023	0.003	0.0741	0.0480	0.1234
glucose	03:49455330	rs11715915	*AMT*	C	T	0.012	0.002	-0.0097	0.0441	0.8259
glucose	03:170717521	rs11920090	*SLC2A2*	T	A	0.026	0.003	-0.0971	0.0626	0.1213
glucose	09:111680359	rs16913693	*IKBKAP*	T	G	0.043	0.007	0.2047	0.0907	0.0246
glucose	11:61571478	rs174550	*FADS1*	T	C	0.019	0.002	0.0536	0.0437	0.2212
glucose	06:7213200	rs17762454	*RREB1*	T	C	0.014	0.002	0.0880	0.0465	0.0591
glucose	07:15064309	rs2191349	*DGKB/TMEM195*	T	G	0.029	0.002	0.0441	0.0423	0.2980
glucose		rs2302593	*GIPR*	C	G	0.014	0.002	0.0588	0.0400	0.1422
glucose	12:56865338	rs2657879	*GLS2*	G	A	0.016	0.003	0.0634	0.0581	0.2757
glucose	01:214159256	rs340874	*PROX1*	C	T	0.013	0.002	0.0073	0.0392	0.8528
glucose	14:100839261	rs3783347	*WARS*	G	T	0.017	0.003	0.0172	0.0504	0.7336
glucose	09:139256766	rs3829109	*LOC728489*	G	A	0.017	0.003	-0.0241	0.0442	0.5859
glucose	10:114756041	rs4506565	*TCF7L2*	T	A	0.021	0.002	-0.0074	0.0455	0.8704
glucose	07:44235668	rs4607517	*GCK*	A	G	0.057	0.003	0.0368	0.0577	0.5237
glucose	05:95539448	rs4869272	*PCSK1*	T	C	0.018	0.002	-0.0147	0.0445	0.7407
glucose	02:169763148	rs560887	*G6PC2*	C	T	0.071	0.002	0.1095	0.0426	0.0105
glucose	13:33554302	rs576674	*KL*	G	A	0.017	0.003	0.0227	0.0627	0.7169

glucose	20:39743905	rs6072275	*TOP1*	A	G	0.016	0.003	-0.0284	0.0534	0.5946
glucose	20:22557099	rs6113722	*FOXA2*	G	A	0.035	0.005	-0.1139	0.1062	0.2842
glucose	07:50791579	rs6943153	*GRB10*	T	C	0.015	0.002	0.0337	0.0425	0.4283
glucose	03:185513392	rs7651090	*IGF2BP2*	G	A	0.013	0.002	0.0609	0.0437	0.1645
glucose	05:76425867	rs7708285	*ZBED3*	G	A	0.015	0.003	0.0683	0.0469	0.1463
glucose	02:27741237	rs780094	*GCKR*	C	T	0.027	0.002	-0.0082	0.0438	0.8517
glucose	09:4292152	rs7867224	*GLIS3*	A	G	0.013	0.002	0.0424	0.0410	0.3018
glucose	11:47336320	rs7944584	*MADD*	A	T	0.023	0.002	0.0630	0.0485	0.1943
glucose	06:20686996	rs9368222	*CDKAL1*	A	C	0.014	0.002	0.0743	0.0428	0.0832
glucose	08:9177732	rs983309	*PPP1R3B*	T	G	0.026	0.003	0.1645	0.0667	0.0142

ESM Table 4 Tests for heterogeneity from the Inverse variance weighted analysis.

Outcome	Cohort	H	I^2
Bone area of total hip	SMCC	1.1	11
Bone area of total hip	PIVUS	1.0	1
Bone area of total hip	ULSAM	1.0	0
BMD of total hip	SMCC	1.0	6
BMD of total hip	PIVUS	1.0	7
BMD of total hip	ULSAM	1.3	40

ESM Table 5. F-statistics for Swedish Mammography Cohort clinical (SMCC), Prospective Investigation in the Vasculature of Uppsala Seniors (PIVUS) and Uppsala Longitudinal Study of Adult Men (ULSAM) from our one sample MR analysis using the weighted genetic risk score (wGRS)

	SMCC	PIVUS	ULSAM
wGRS	195.50493	40.103464	50.275994

Adjusted for age and genetic principle components.

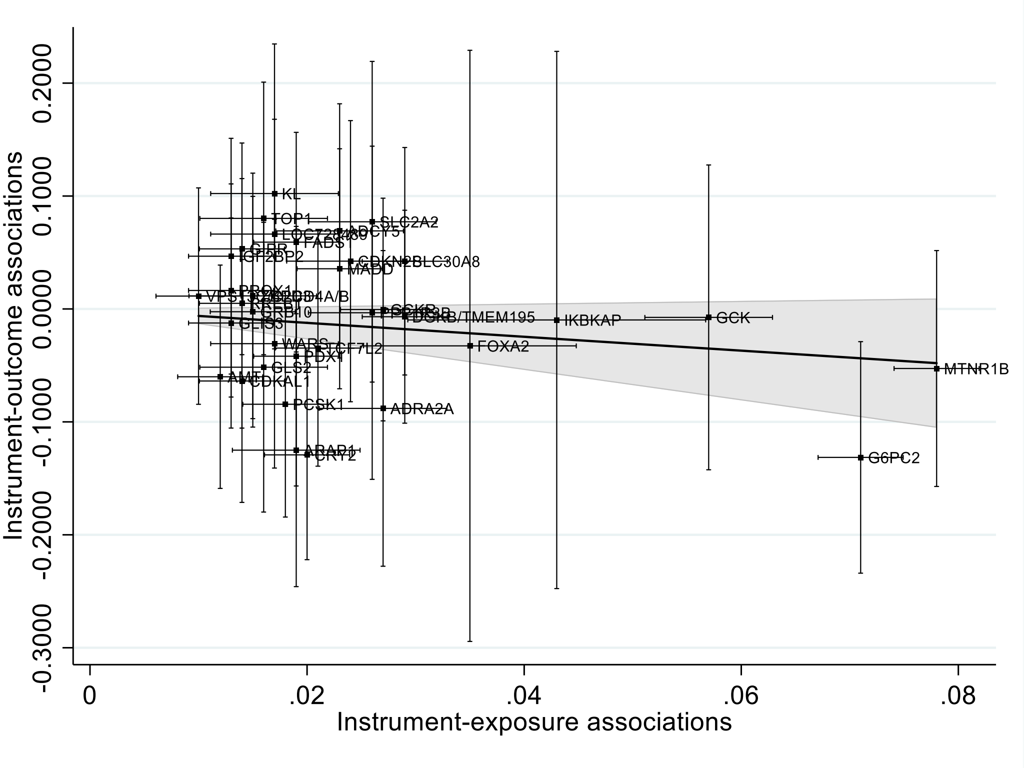

ESM Fig. 1 The association of 35 glucose associated SNPs with bone area measured in SMCC

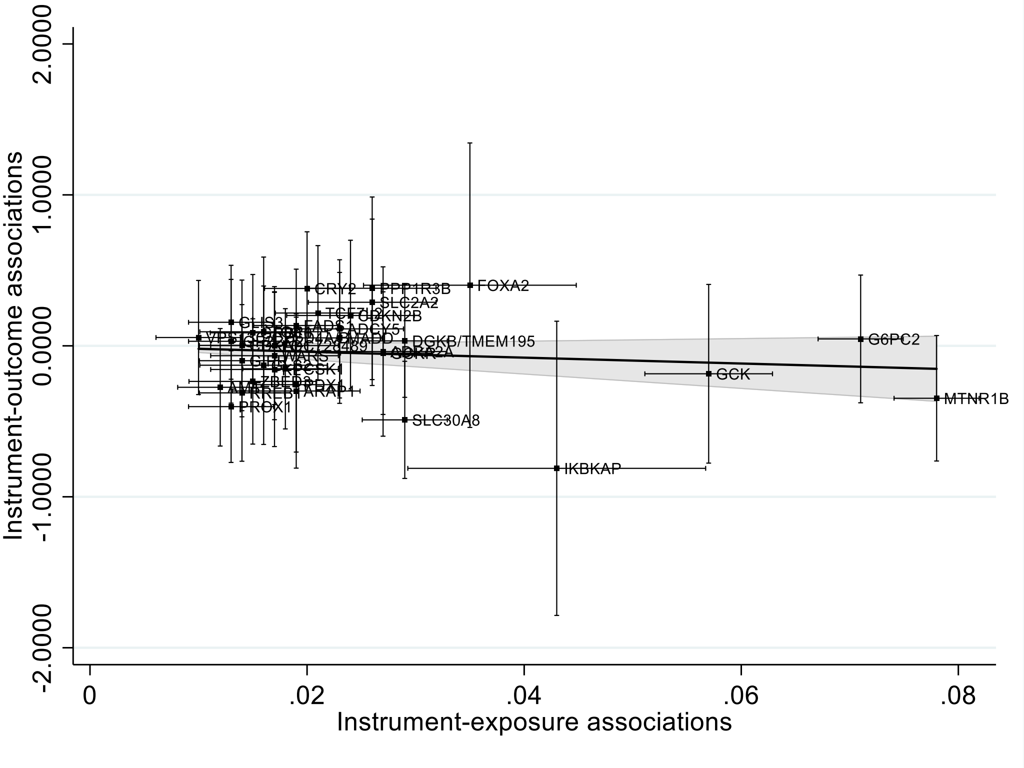

ESM Fig. 2 The association of 35 glucose associated SNPs with bone area measured in PIVUS

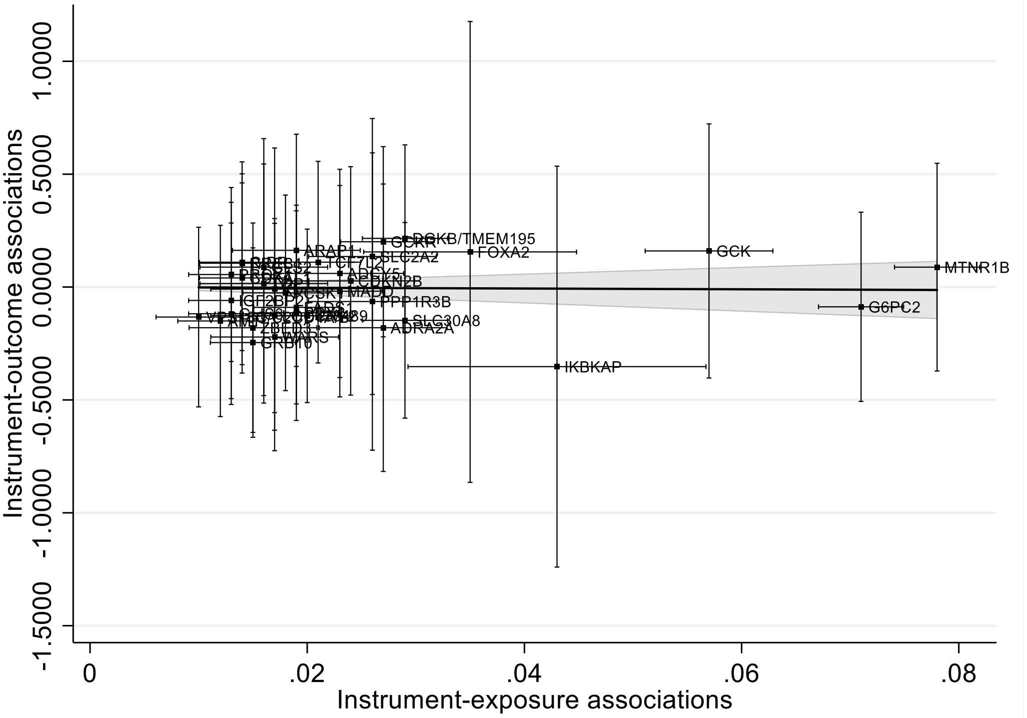

ESM Fig. 3 The association of 35 glucose associated SNPs with bone area measured in ULSAM

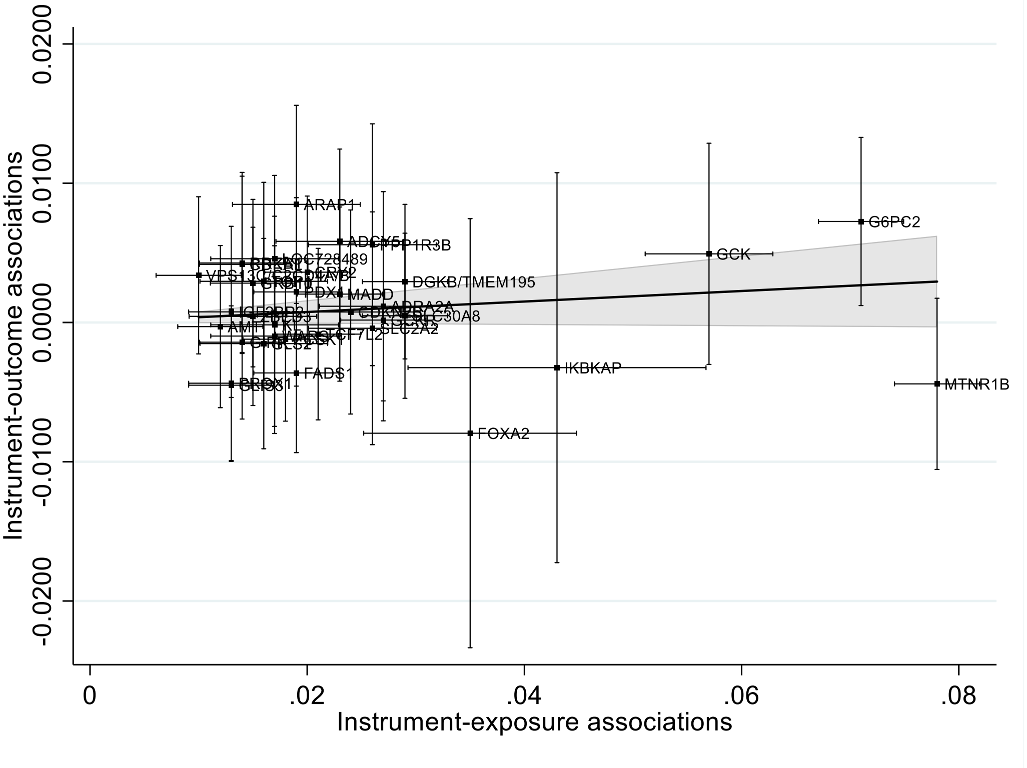

ESM Fig. 4 The association of 35 glucose associated SNPs with bone mineral density (BMD) measured in SMCC

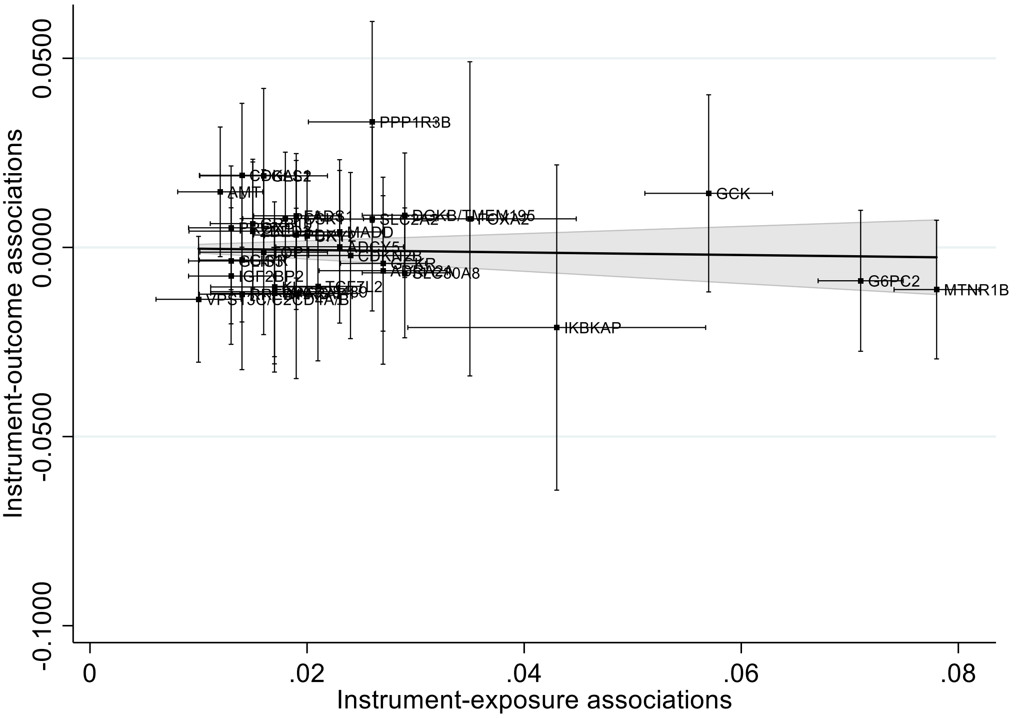

ESM Fig. 5 The association of 35 glucose associated SNPs with bone mineral density (BMD) measured in PIVUS

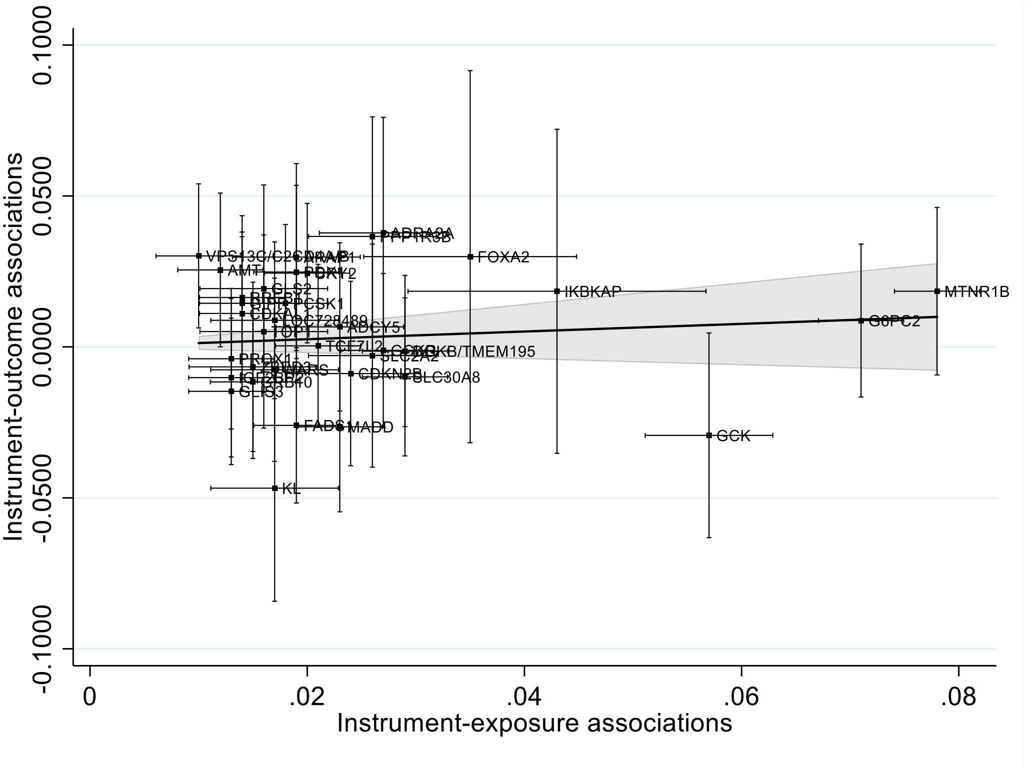

ESM Fig. 6 The association of 35 glucose associated SNPs with bone mineral density (BMD) measured in ULSAM

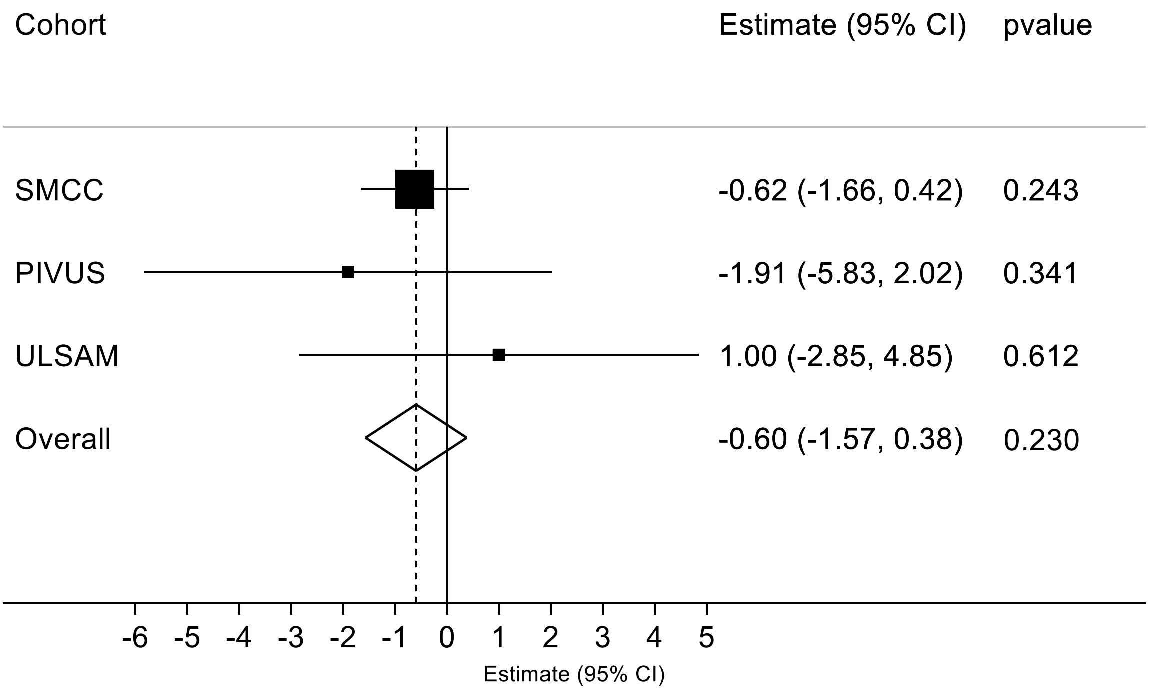

ESM Fig. 7 Meta-analysis of glucose variants to bone area using the weighted median approach in SMCC, PIVUS & ULSAM

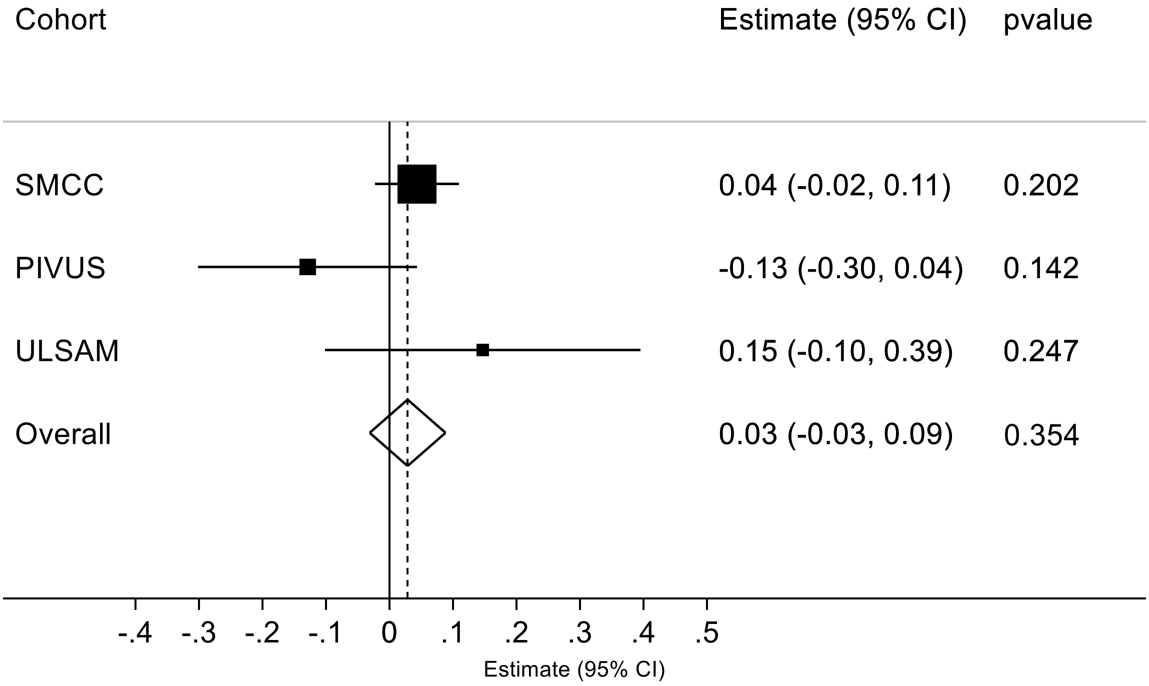

ESM Fig. 8 Meta-analysis of glucose variants to bone mineral density (BMD) using the weighted median approach in SMCC, PIVUS & ULSAM

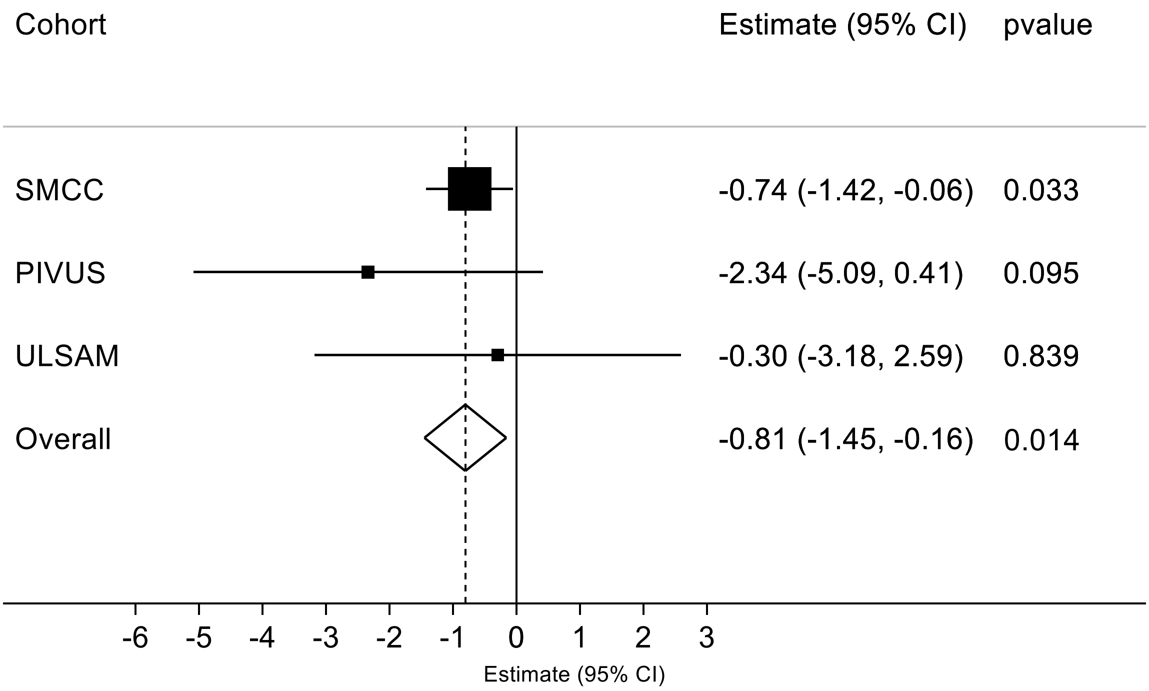

ESM Fig. 9 Meta-analysis of glucose variants to bone area adjusted for the genetic effect of height in SMCC, PIVUS & ULSAM

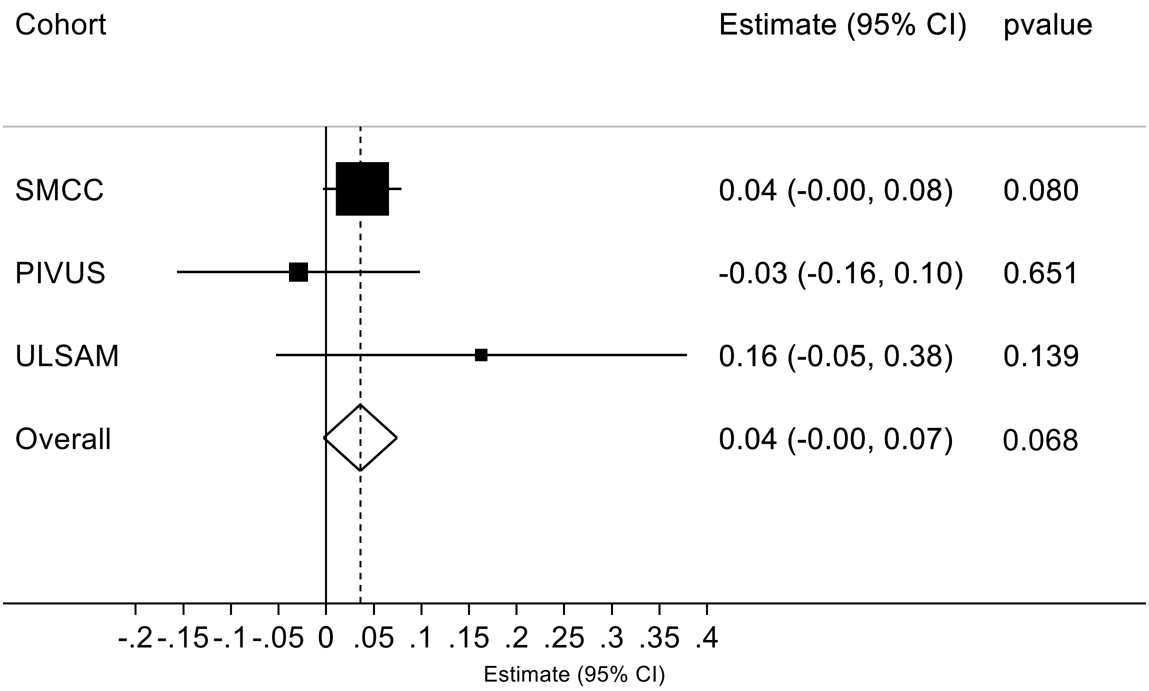

ESM Fig. 10 Meta-analysis of glucose variants to bone mineral density adjusted for the genetic effect of height in SMCC, PIVUS & ULSAM

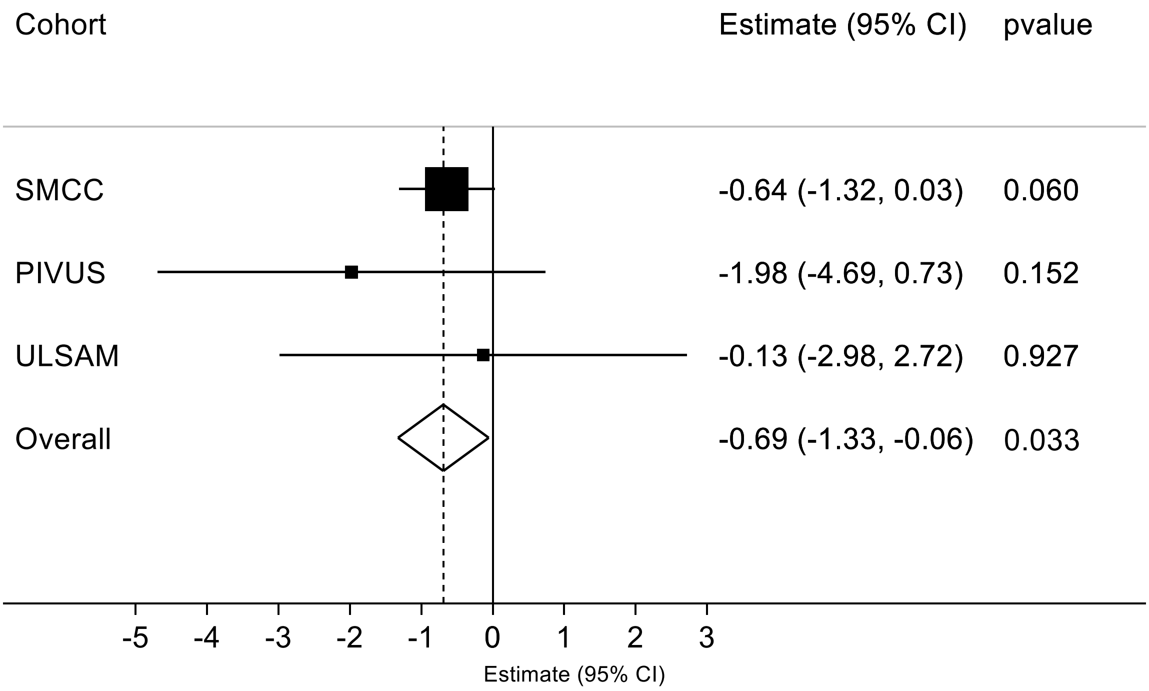

ESM Fig. 11 Meta-analysis of glucose variants to bone area after removing SNP (rs7651090, for gene IGF2BP2) in SMCC, PIVUS & ULSAM

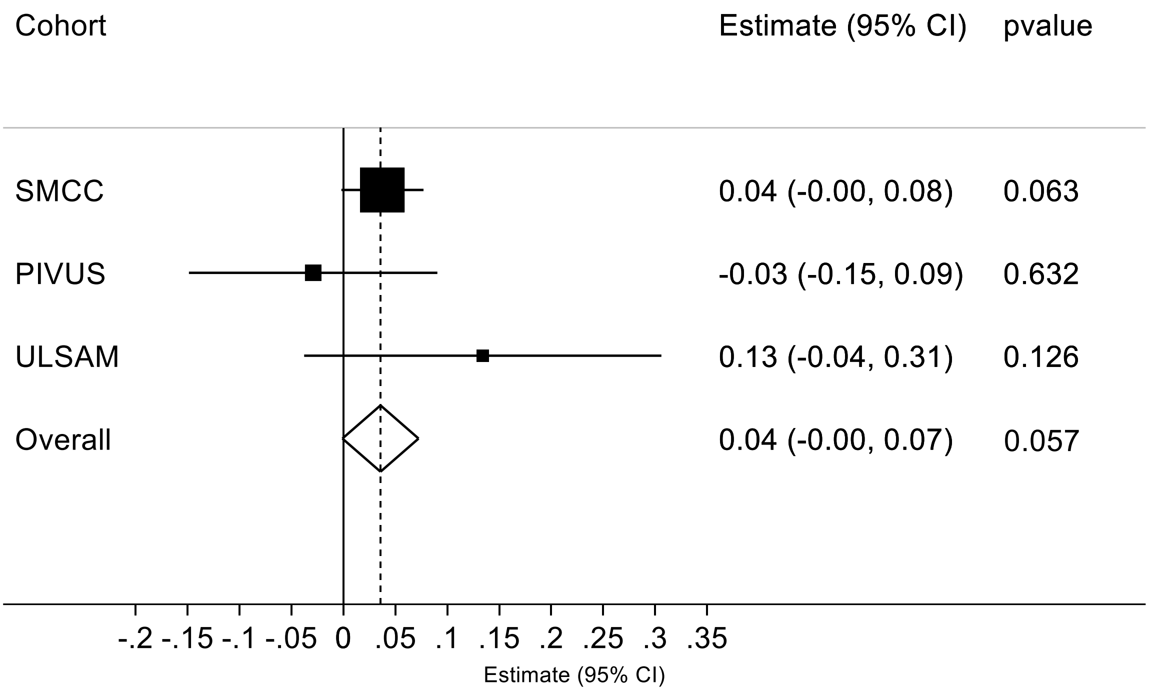

ESM Fig. 12 Meta-analysis of glucose variants to bone mineral density after removing SNP (rs7651090, for gene IGF2BP2) in SMCC, PIVUS & ULSAM

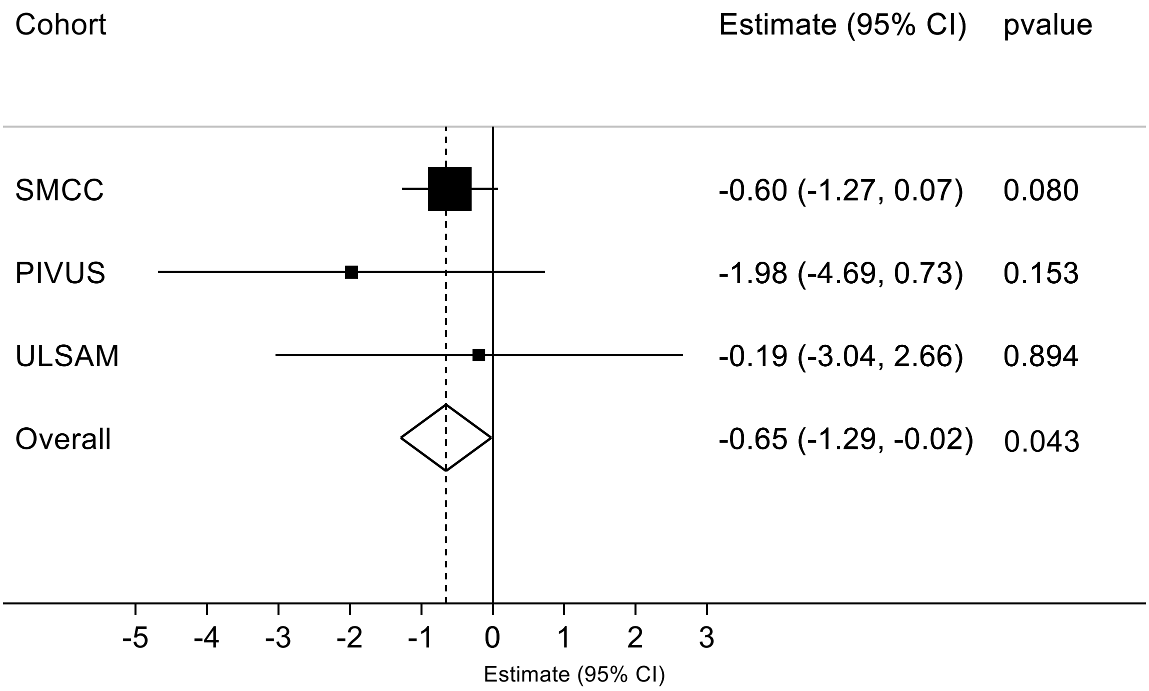

ESM Fig. 13 Meta-analysis of glucose variants to bone area adjusted for the genetic effect of BMI in SMCC, PIVUS & ULSAM

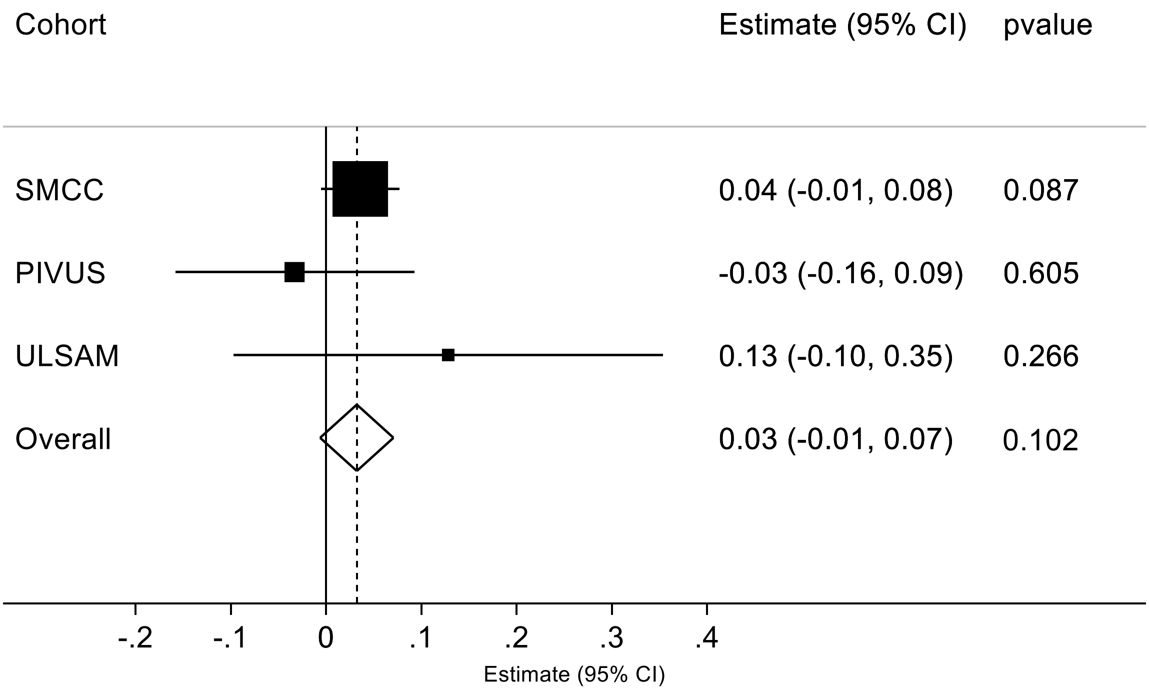

ESM Fig. 14 Meta-analysis of glucose variants to bone mineral density adjusted for the genetic effect of BMI in SMCC, PIVUS & ULSAM

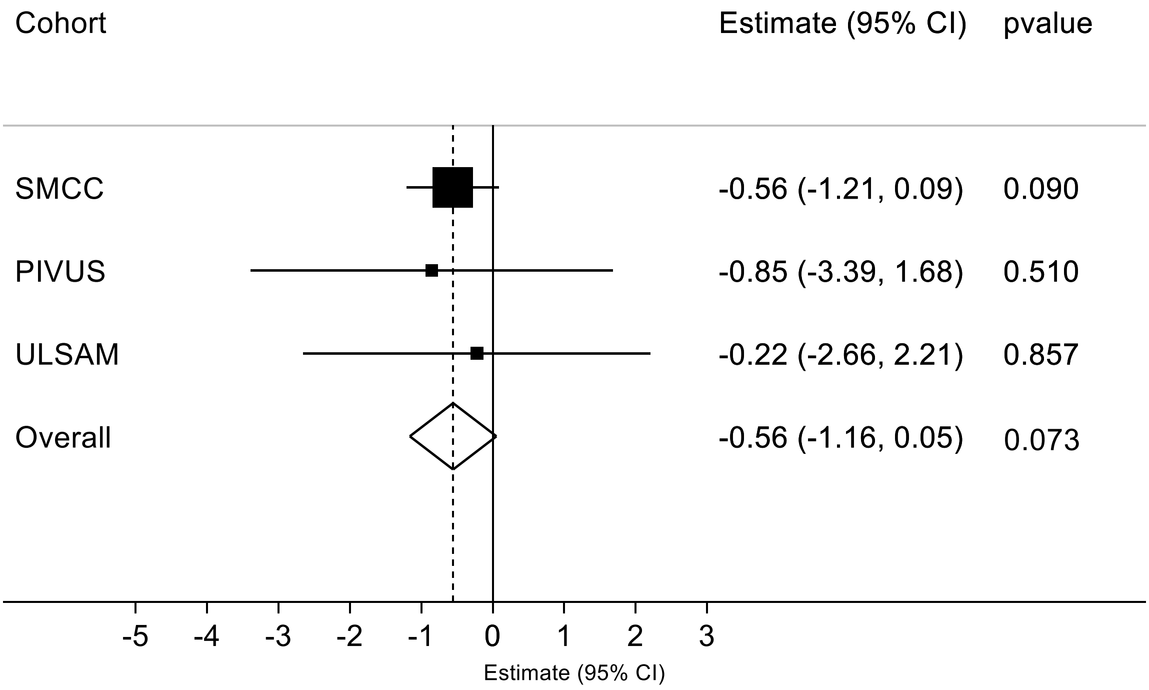

ESM Fig. 15 Meta-analysis of glucose variants to bone area in SMCC, PIVUS & ULSAM including those with T2DM

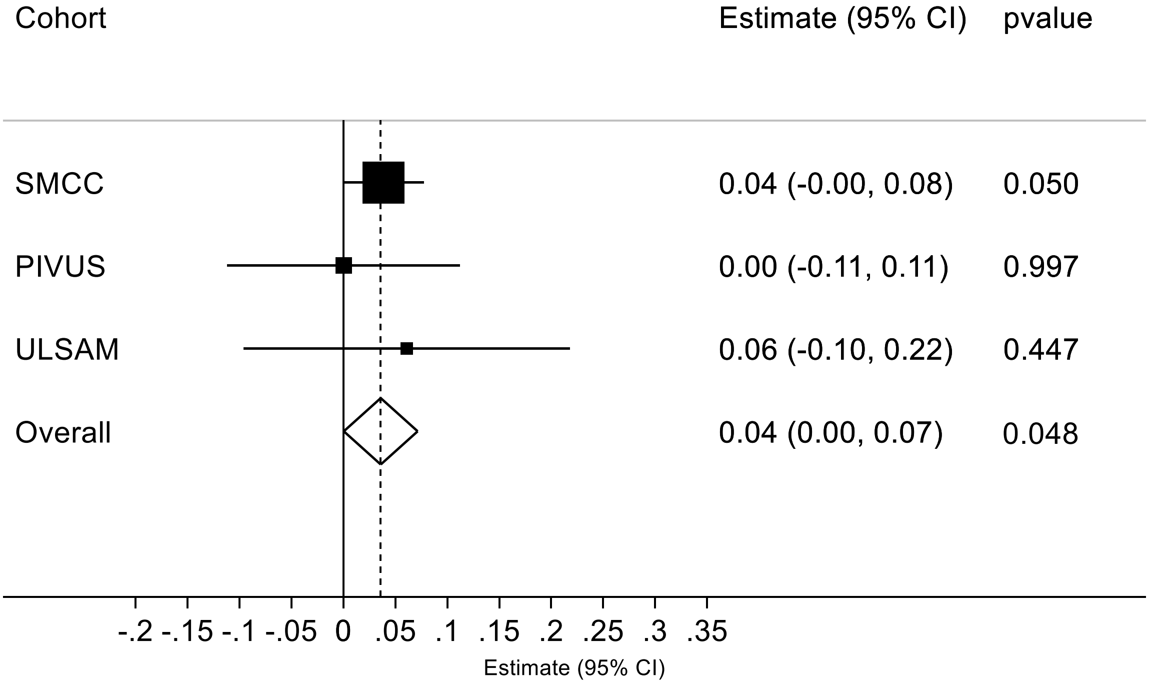

ESM Fig. 16 Meta-analysis of glucose variants to BMD in SMCC, PIVUS & ULSAM including those with T2DM

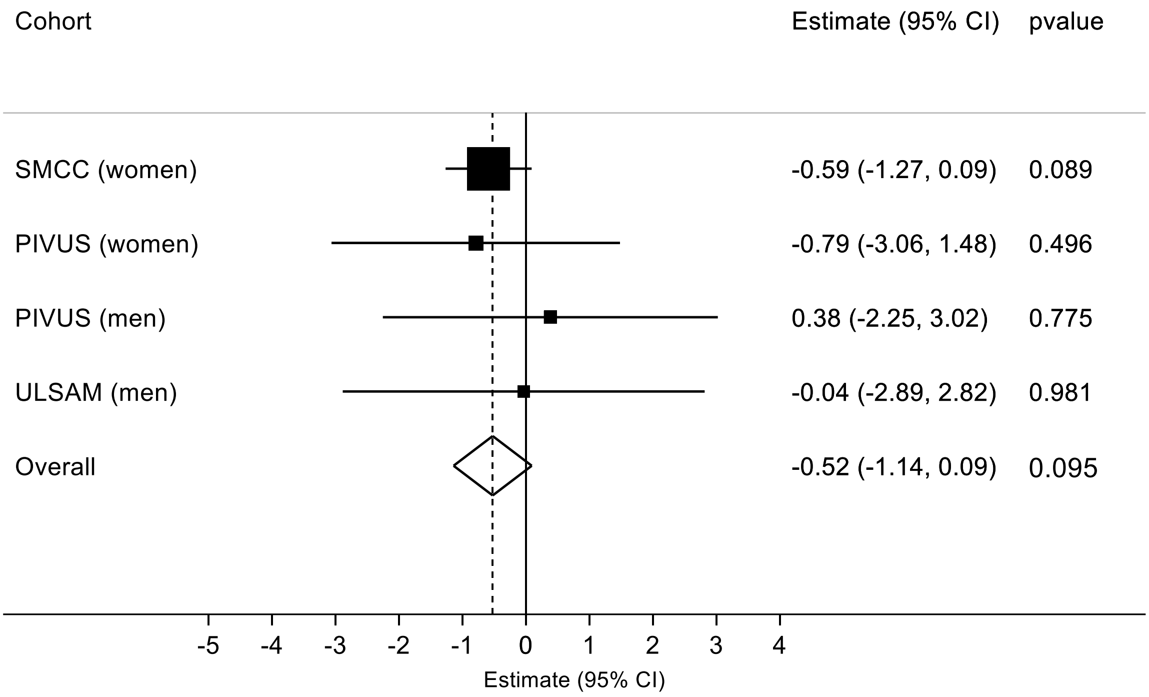

ESM Fig. 17 Meta-analysis of glucose variants to bone area in SMCC, PIVUS & ULSAM using sex specific beta estimates for fasting glucose (https://www.magicinvestigators.org/downloads/)

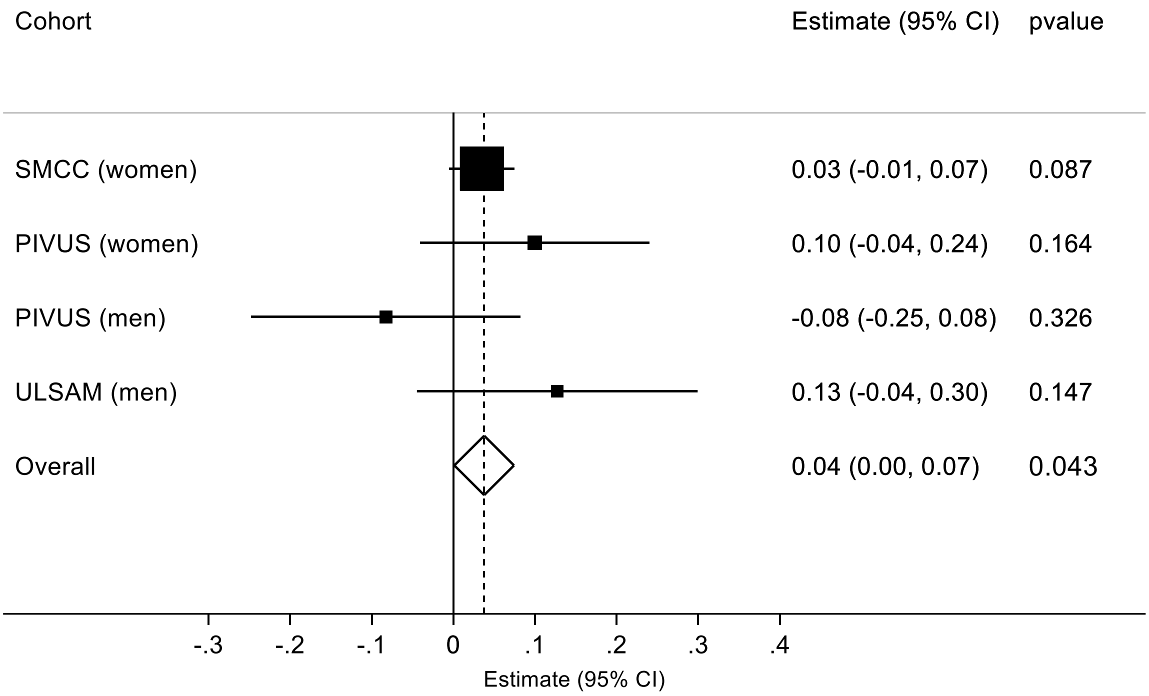

ESM Fig. 18 Meta-analysis of glucose variants to BMD in SMCC, PIVUS & ULSAM using sex specific beta estimates for fasting glucose (https://www.magicinvestigators.org/downloads/)

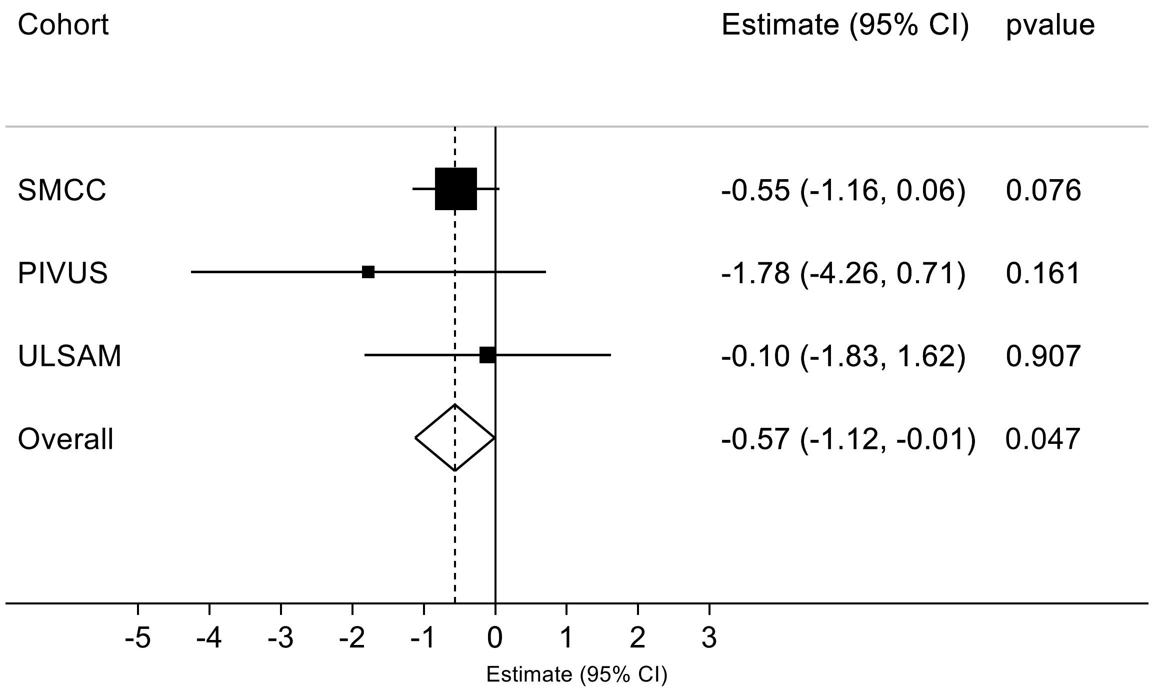

ESM Fig. 19 Meta-analysis of weighted genetic risk score (wGRS) to bone area in SMCC, PIVUS & ULSAM

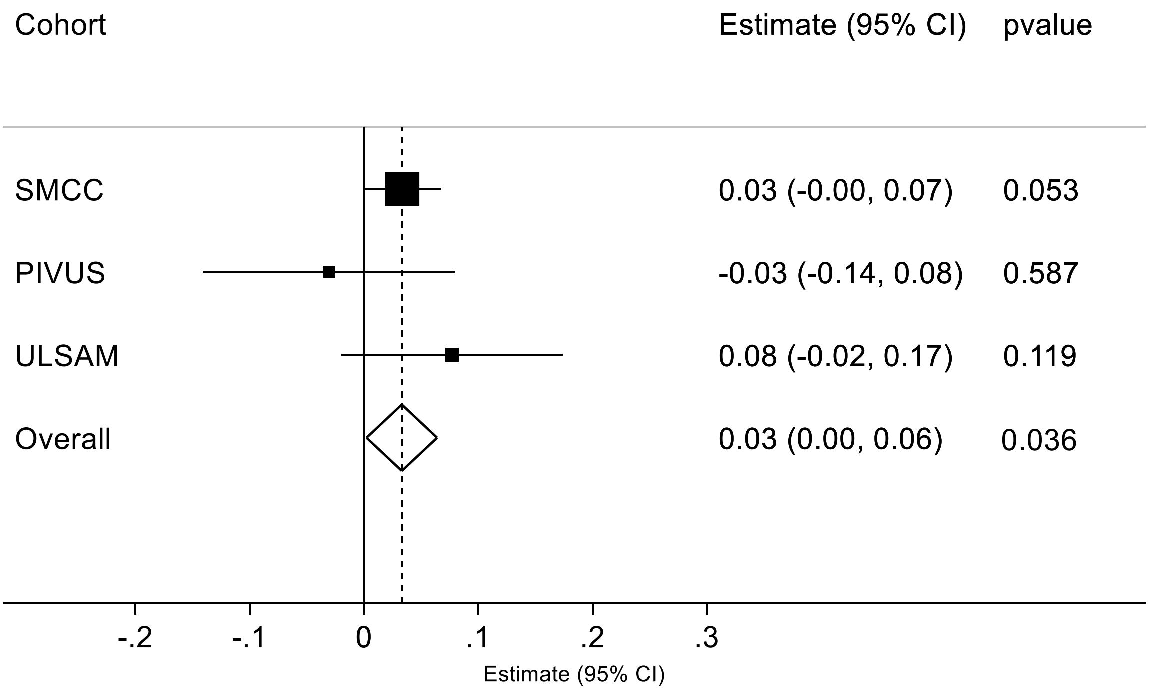

ESM Fig. 20 Meta-analysis of weighted genetic risk score (wGRS) to bone mineral density (BMD) in SMCC, PIVUS & ULSAM

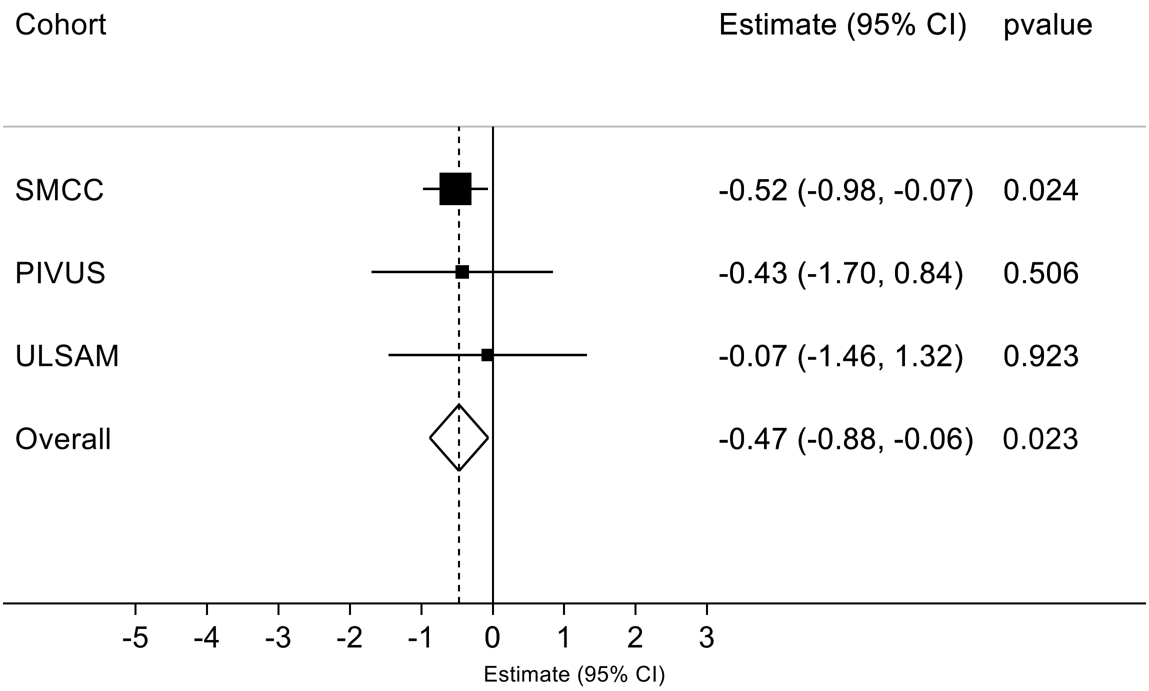

ESM Fig. 21 Meta-analysis of weighted genetic risk score (wGRS) to bone area adjusted for height and BMI in SMCC, PIVUS & ULSAM

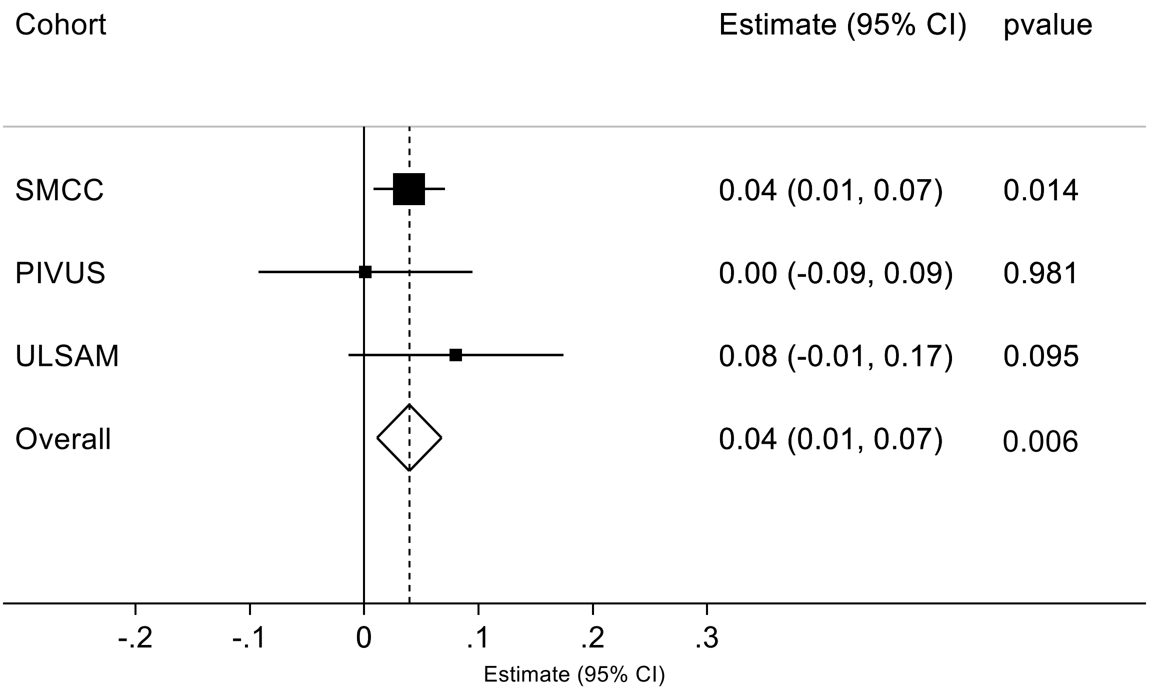

ESM Fig. 22 Meta-analysis of weighted genetic risk score (wGRS) to bone mineral density (BMD) adjusted for height and BMI in SMCC, PIVUS & ULSAM
